# Rotaxanes with dynamic mechanical chirality: Systematic studies on synthesis, enantiomer separation, racemization, and chiral-prochiral interconversion

**DOI:** 10.3389/fchem.2022.1025977

**Published:** 2022-10-28

**Authors:** Fumitaka Ishiwari, Toshikazu Takata

**Affiliations:** ^1^ Department of Chemical Science and Engineering, Tokyo Institute of Technology, Meguro-ku, Japan; ^2^ School of Materials and Chemical Technology, Tokyo Institute of Technology, Yokohama, Japan; ^3^ Graduate School of Advanced Science and Engineering, Hiroshima University, Hiroshima, Japan

**Keywords:** dynamic mechanical chirality, rotaxane, mechanical chirality, prochiral, racemization, chiral-prochiral interconversion, enantiomer separation

## Abstract

Dynamic mechanical chirality of [2]rotaxane consisting of a *C*_s_ symmetric wheel and a *C*_2v_ symmetric axle is discussed *via* the synthesis, enantiomer separation, racemization, and chiral-prochiral interconversion. This [2]rotaxane is achiral and/or prochiral when its wheel locates at the center of the axle, but becomes chiral when the wheel moves from the center of the axle. These were proved by the experiments on the enantiomer separation and racemization. The racemization energy of the isolated single enantiomers was controlled by the bulkiness of the central substituents on the axle. Furthermore, the chiral-prochiral interconversion was achieved by relative positional control of the components. The present systematic studies will provide new insight into mechanically chiral interlocked compounds as well as the utility as dynamic chiral sources.

## Introduction

**FIGURE 1:**
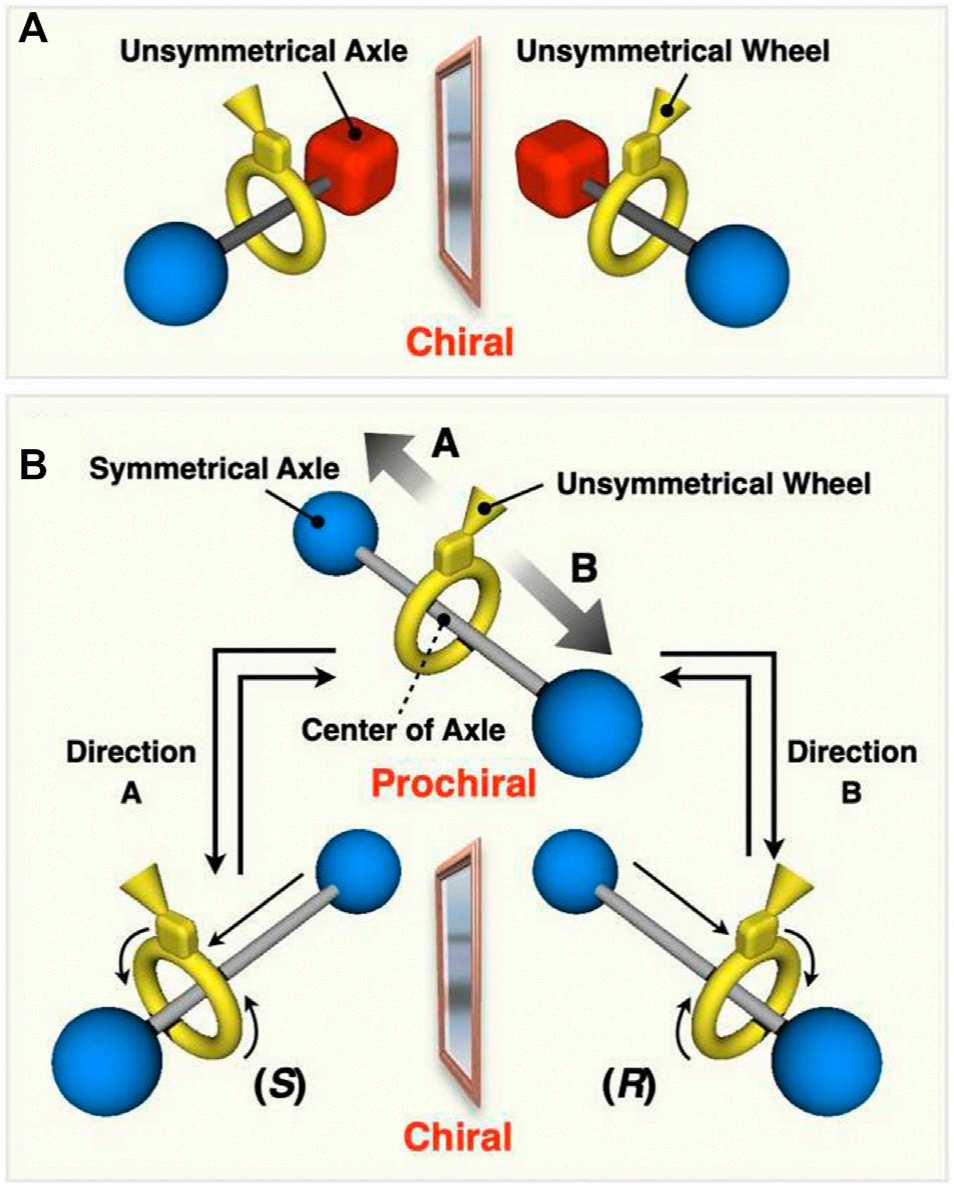
**(A)** Mechanically chiral [2]rotaxane consisting of an unsymmetrical wheel and an symmetrical axle. **(B)** Generation of co-conformational dynamic mechanical chirality in [2]rotaxane consisting of a *C*_s_ symmetric wheel and a *C*_2v_ symmetric axle.

**FIGURE 2:**
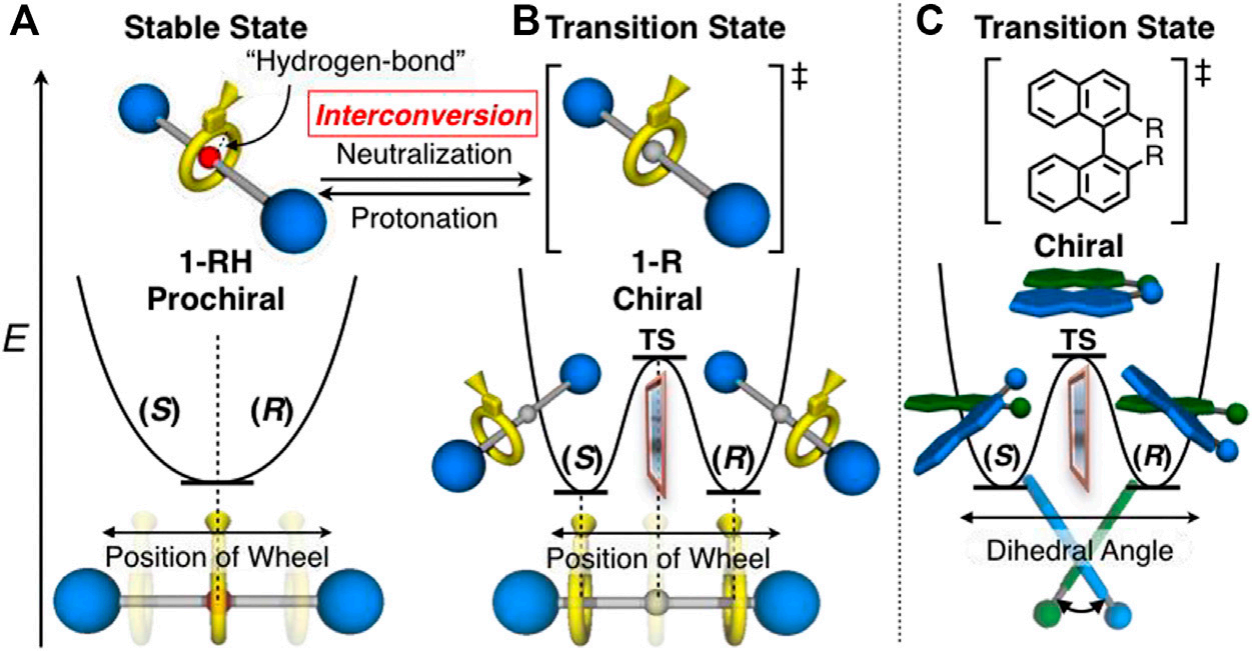
Chiral-prochiral interconversion and energy diagrams of **(A) 1-RH**, **(B) 1-R** (see also Scheme 1 and Figure 5A and Supplementary Figure S50) and **(C)** axially chiral 1,1′-binaphtyl derivatives for comparison.

Rotaxanes derived by combination of symmetrical and unsymmetrical components can generate chirality due to an intramolecular restriction. If the microscopic conformation and co-conformation of the component are not considered, [2]rotaxane consisting of at least one symmetrical component has no chirality, while a [2]rotaxane consisting of two unsymmetrical components actually has chirality, *i.e.* mechanically planar chirality (Figure 1A) (representative accounts and reviews; Sauvage and Dietrich-Buchecker, 1999; Sauvage, 1990; Forgan et al., 2011, Neal and Goldup, 2014, Jamieson et al., 2018, Maynard and Goldup, 2020). Pioneering works by Sauvage (Mitchell and Sauvage, 1988, Dietrich-Buchecker and Sauvage et al., 1989; Kaida et al., 1993) and Vögtle (Reuter et al., 2000a; Reuter et al., 2000b; Reuter et al., 2001; Yamamoto et al., 1997; Jäger et al., 1996; Vögtle et al., 2001; Lukin et al., 2003; Lukin and Vögtle, 2005) on the synthesis and enantiomer separation of mechanically or topologically chiral rotaxanes and catenanes were followed by our enantiomer separation and asymmetric synthesis of simple rotaxanes (Makita et al., 2007) and by Goldup’s extensive works (Bordoli and Goldup, 2014; de Juan et al., 2022). Kametani and co-workers suggested the chiral recognition ability of mechanically planar rotaxane (Kameta et al., 2006). Recently, we also showed the effectiveness of mechanically chiral compound as chiral sources to induce one-handed helicity to polyacetylenes (Ishiwari et al., 2017). More recently, Kawabata and co-workers have reported the efficient synthesis of optically active mechanically planar chiral rotaxane with by kinetic resolution strategy (Imayoshi et al., 2020). Goldup and co-workers also have demonstrated that the chiral interlocking auxiliary strategy for the synthesis of mechanically planar chiral rotaxanes (de Juan et al., 2022). Taking dynamic nature of the components into account, it is expected that the rotaxane shown in Figure 1B would become chiral, because the movement of the wheel from the center of the axle (when the axle is fixed) would make the originally symmetrical axle unsymmetrical (Figures 1B, 2). The chirality in the rotaxanes of this type is now classified as co-conformationally mechanically planar chiral rotaxane (Jamieson et al., 2018). Due to such co-conformational behaviors, interlocked compounds exhibit various chirality. As a pioneering work, Stoddart and co-workers reported the generation of co-conformational helical chirality in catenates (Vignon et al., 2004). Recently, Cougnon and co-workers reported similar diastereomeric amplification of a co-conformationally mechanically chiral [2]catenane (Caprice et al., 2021). More recently, Goldup and co-workers have synthesized a co-conformationally chiral catenane (Rodríguez-Rubio et al., 2022). When the barrier to co-conformational motion of the rotaxane in Figure 1B is low enough, such rotaxanes can express a “dynamic mechanical chirality” produced from dynamic nature of mechanical bond, that is, similar to the chirality of axially chiral binaphthyls, since they also lose their chirality when the two arene moieties align coplanar in the transition state (Figure 2C). Thus, the present rotaxane shown in Figure 1B can become co-conformationally chiral (Figure 2B) or achiral (prochiral) (Figure 2A) depending completely on the relative position of the components. This means that for this type of dynamically chiral [2]rotaxane, racemization (*i*.*e*. mechanostereoinversion) occurs by the movement of wheel from one side to the other side (translational movement), and chiral-prochiral interconversion can be possible by controlled positional switching of components of rotaxane. Development of such new class of mechanically chiral rotaxane will provide new insights into chiral science. It has been reported that [3]rotaxanes consisting of two unsymmetrical wheels and one symmetrical axle generate mechanical chirality, but they do not undergo the racemization and do not become prochiral (Schmieder et al., 1999; Kishan et al., 2006). Mechanically point-chiral rotaxanes with chemically symmetric axles reported by Leigh et al. are essentially different class of chiral species from the ones with dynamic mechanical chirality (Alvarez-Pérez et al., 2009; Cakmak et al., 2016). Recently, Saito et al., reported the studies on synthesis, enantiomer separation, racemization of dynamically chiral [2]rotaxanes (Mochizuki et al., 2017), but the isolation of prochiral species would be difficult. Credi et al. reported an asymmetric induction of dynamically chiral [2]rotaxanes (Corra et al., 2019), but the isolation of optically active rotaxanes with purely mechanical chirality would be difficult, which will prevent the studies on the enantiomer separation and racemization behaviors, and future utilizations as chiral sources. Thus, there is much room for further detailed investigation in this class of compounds with dynamic mechanical chirality; for example, combined utilization with switching function, and chiral-prochiral interconversion behavior. We have independently investigated this type of co-conformationally mechanically planar chiral rotaxanes with dynamic chirality having smaller molecular weights, and achieved the isolation of the prochiral species and chiral-prochiral interconversion. Here we report the systematic studies of the co-conformationally mechanically planar chiral rotaxanes with dynamic chirality, including the synthesis of a series of dynamically chiral rotaxanes with various substituents at the center of the axle, evaluation of their racemization behavior, and chiral-prochiral interconversion by the switching function.

## Results and discussion

### Molecular Design

**SCHEME 1:**
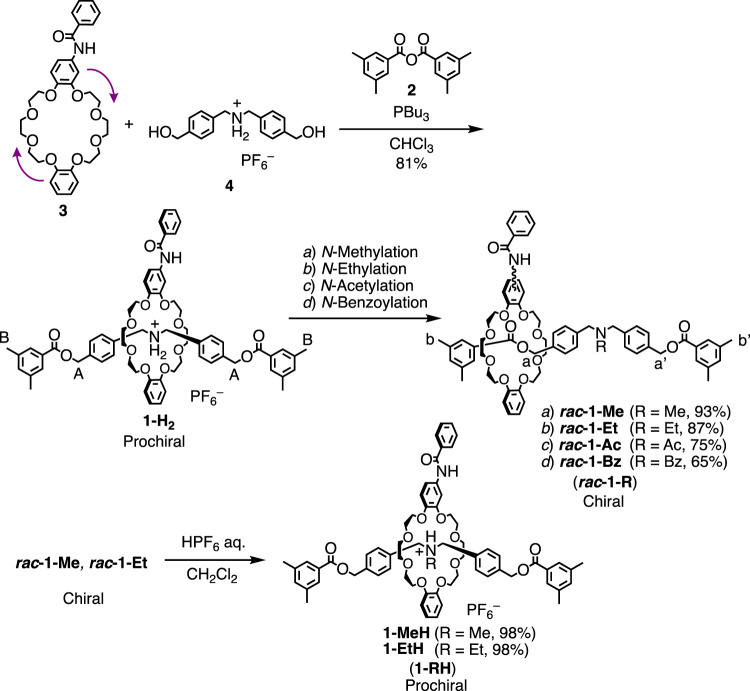
Synthesis of **1-H_2_**, ***rac*-1-R**s, and **1-RH** (See the Supplementary Materials for details).

In this study, we designed [2]rotaxanes **1-H**_**2**_, ***rac*-1-R** and ***rac*-1-RH** consisting of a *C*_s_-symmetric crown ether-type wheel and a symmetrical axles shown in Scheme 1. Since the *sec*-ammonium group of **1-H**_**2**_ can serve as a quite efficient station of the wheel to stop its translational motion on the axle (Cao et al., 2000; Kihara et al., 2000; Nakazono and Takata, 2010), the wheel component of [2]rotaxane **1-H**_**2**_ should be strongly localized on the center of axle as shown in Figure 2A. Therefore, [2]rotaxane **1-H**_**2**_ should be prochiral. The movement of the wheel from the central *sec*-ammonium group by direct neutralization of the *sec*-ammonium group is hardly possible due to the extremely strong hydrogen bonding between *sec*-ammonium group and crown ether by intramolecular proximity and size effect on complexation (Cao et al., 2000; Kihara et al., 2000; Nakazono and Takata, 2010). Thus, in order to move the wheel from the center of axle, we applied the two chemical modification techniques, acylation (Kihara et al., 2000; Tachibana et al., 2006; Kihara et al., 2007) and reductive alkylation (Nakazono et al., 2008; Suzuki et al., 2010; Ishiwari et al., 2011; Suzuki et al., 2012), to the *sec*-ammonium group of **1-H**_**2**_ to introduce the steric barrier on the center of axle (Scheme 1, Figure 2B). Our group previously reported that acylation reaction to the *sec*-ammonium group of this type of rotaxanes efficiently underwent to desymmetrize the chemically symmetrical axle component (Suzuki et al., 2010). In this study, we employed acetyl group and bulkier benzoyl group because it is reported that acetyl group is bulky enough to stop the translational motion of the wheel on this type of axle (Suzuki et al., 2010). The acylation reactions to prochiral **1-H**_**2**_ should afford chiral rotaxane **1-Ac** and **1-Bz** (Scheme 1, Figure 2B). However, these rotaxanes with acyl groups lack the switchability of the position of the wheel, and in turn are not capable of chiral-prochiral interconversion. Thus, we decided to introduce alkyl group to **1-H**_**2**_ by reductive *N*-alkylation reaction in order to move the wheel component from the center of axle and to endow the rotaxane with the switchability of the position of the wheel by protonation and deprotonation of the *tert*-amine moiety (Nakazono et al., 2008; Suzuki et al., 2010; Ishiwari et al., 2011; Suzuki et al., 2012). In the neutral form (**1-R**, Figure 2A), the rotaxane will behave as chiral molecules by the steric barrier by the introduced alkyl group (Scheme 1, Figure 2B). In the protonated form (**1-RH**), if the wheel can be localized on the central *tert*-ammonium moiety, the protonated rotaxane **1-RH** will behave as prochiral entity (Scheme 1, Figure 2A). Therefore, protonation and deprotonation of the *tert*-amine moiety will give rise to chiral prochiral interconversion (Figures 2A,B). Since we have no information on the steric barriers caused by *N*-alkyl group on the axle, in this study, we introduced relatively small methyl and ethyl groups (**1-Me** and **1-Et**, Scheme 1) so that the wheel component can overcome and localized the introduced *N*-alkyl group.

### Synthesis and Characterizations

Aforementioned rotaxanes were synthesized as shown in Scheme 1. According to our typical synthetic protocol for a similar crown ether-based rotaxane (Kawasaki et al., 1999), a symmetrical *sec*-ammonium salt axle (**4**) having two hydroxyl groups at the termini and a mono-substituted dibenzo-24-crown ether (DB24C8) (**3**) were mixed and treated with 3,5-dimethylbenzoic anhydride (**2**) as a terminal stopper for the axle in the presence of tributylphosphine as a catalyst to give the corresponding rotaxane (**1-H**_**2**_) in 81% yield (See Supplementary Materials for details). By treating **1-H**_**2**_ with Ac_2_O or BzCl in the presence of Et_3_N in DMF, corresponding *N*-acylated rotaxanes ***rac*-1-Ac** (75%) and ***rac*-1-Bz** (65%) respectively in good yields (See Supplementary Materials for details) (Tachibana et al., 2006; Kihara et al., 2007). *N*-Methylation reaction (Nakazono et al., 2008; Suzuki et al., 2010; Ishiwari et al., 2011; Suzuki et al., 2012) of **1-H**_**2**_ was carried out by reactive *N*-methylation using paraformaldehyde and NaBH(OAc)_3_ in the presence of Et_3_N in *N*-methylpyrrolidone (NMP) at room temperature. The purification by Al_2_O_3_ column chromatography arrowed the isolation of the corresponding *N*-methylated rotaxane ***rac*-1-Me** in an excellent yield (93%). *N*-Ethylation reaction (Suzuki et al., 2010) of **1-H**_**2**_ was conducted by reductive *N*-alkylation using NaBH(OAc)_3_ in the presence of Et_3_N in NMP at 70°C to give the corresponding *N*-ethylated rotaxane ***rac*-1-Et** in an excellent yield (87%). In this *N*-ethylation reaction, we did not use the corresponding aldehyde-source because the NaBH(OAc)_3_ at high temperature generates acetaldehyde *in situ* by self-reduction (Suzuki et al., 2010). Protonation of ***rac*-1-Me** and ***rac*-1-Et** quickly proceeded by washing with HPF_6_ aq. to afford the corresponding protonated rotaxanes **1-MeH** and **1-EtH** in excellent yields (98%). The structures of all new compounds were unambiguously characterized by ^1^H, ^13^C NMR, FT-IR spectroscopy, and high-resolution mass spectrometry (Supplementary Figures S1−S32). For N-Acylated compounds (**1-Ac** and **1-Bz**).

### Chiral Structures of Rotaxanes

**FIGURE 3:**
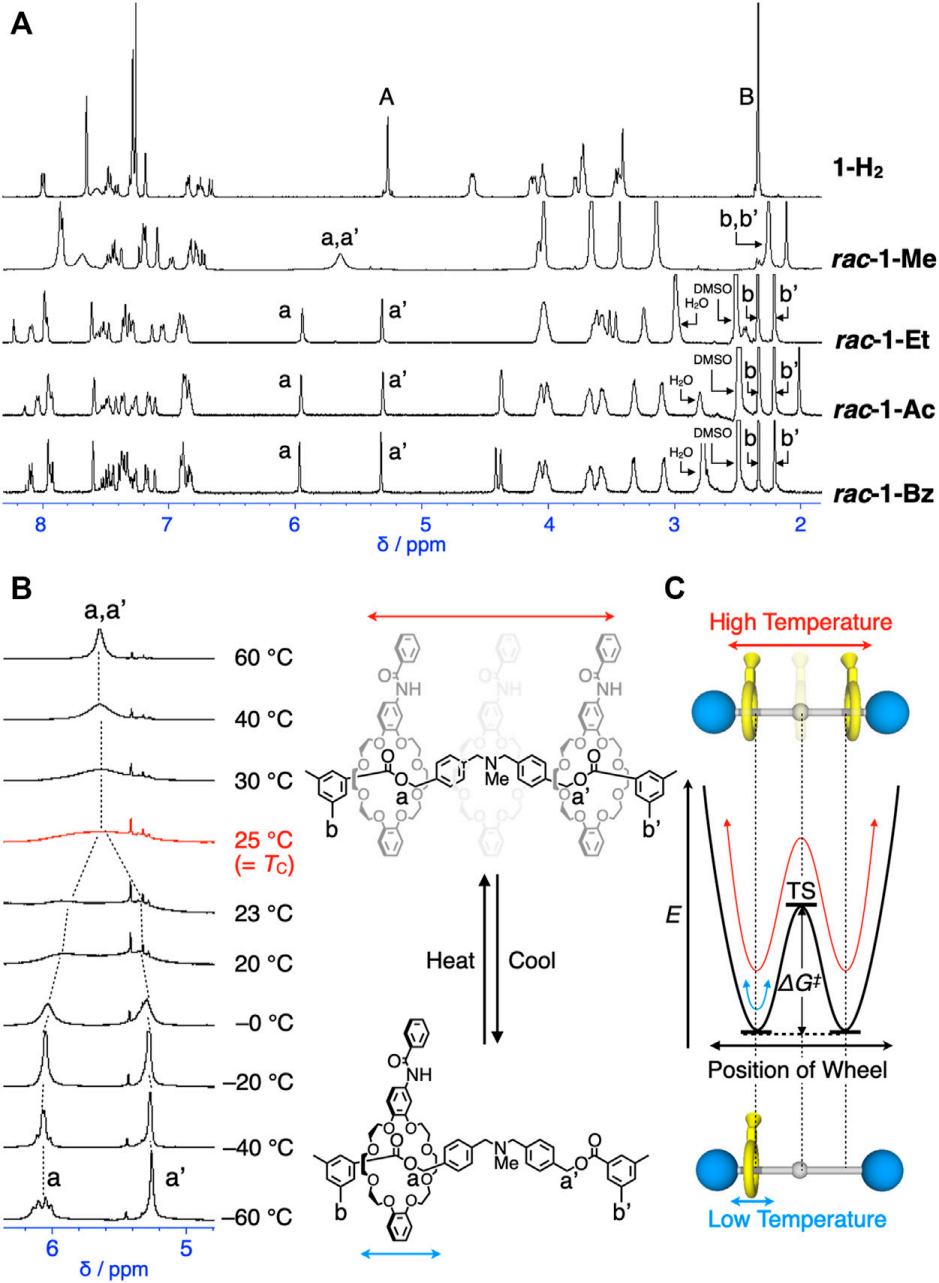
**(A)** ^1^H NMR spectra of **1-H**_**2**_, ***rac*-1-Me**, ***rac*-1-Et**, ***rac*-1-Ac** and ***rac*-1-Bz** (400 MHz, in CDCl_3_ at 298 K for **1-H**_**2**_ and at 333 K, ***rac*-1-Me**, and in DMSO-*d*_6_ at 373 K for ***rac*-1-Et**, at 417 K for ***rac*-1-Ac** and ***rac*-1-Bz**, see also Scheme 1 for the assignments of protons). **(B)** Partial VT-^1^H NMR spectra of ***rac*-1-Me** (400 MHz, CDCl_3_, 213–333 K). **(C)** Mobility and energy diagram of ***rac*-1-Me**.

In the ^1^H and ^13^C NMR spectra of **1-H**_**2**_, all the signals of the axle component appeared symmetrically (Figure 3A, Supplementary Figures S1–S3, Supplementary Figures S21–S25), indicating that the wheel of **1-H**_**2**_ strongly localized on the center of the axle as expected (Kihara et al., 2007). Thus, the structure of **1-H**_**2**_ is not chiral but prochiral. This isolable prochiral 1-H_2_ is an interesting species because such prochiral species is generated only in transition state and cannot be isolated in axially chiral compounds (Figure 2C). Prior to the enantiomer separation of ***rac*-1-R28**s (**1-Me**, **1-Et**, **1-Ac**, and **1-Bz**) by chiral HPLC, we studied the NMR spectra of these rotaxanes. In the ^1^H and ^13^C NMR spectra of ***rac*-1-Et**, ***rac*-1-Ac** and ***rac*-1-Bz**, the NMR signals of the axle components appeared unsymmetrically even at 140°C (Figure 3A, Supplementary Figures S11–S13, Supplementary Figures S17–S25), while those of ***rac*-1-Me** appeared symmetrically at 25°C (Figures 3A,B). Representatively, the signals of *O*-benzylic protons (a and a’, see also Scheme 1) and terminal benzylic protons (b and b’, see also Scheme 1) on the axle components of **1-Et**, **1-Ac**, and **1-Bz** appeared non-equivalently (Figure 3A, Supplementary Figures S11–S13, Supplementary Figures S17–S25). These observation in ^1^H NMR spectra of the rotaxanes, except for rac-1-Me, clearly suggested the wheel components of **1-Et**, **1-Ac**, and **1-Bz** do not undergo fast shuttling with overcoming the central *N*-substituent groups because the steric barriers of the *N*-substituent groups (Et, Ac, and Bz) for the free translation on axle are sufficiently high. Thus, the possibilities of enantiomer separation of enantiomers of **1-Et**, **1-Ac**, and **1-Bz** were suggested.

Due to the low steric barrier by *N*-methyl group of ***rac*-1-Me**, attempted enantiomer separation of ***rac*-1-Me** by chiral HPLC was actually unsuccessful. Thus, we evaluated the thermodynamic parameters of the translational movement, in other words, mechanostereoinversion, *E*, *ΔG* ^‡^, *ΔS* ^‡^ and *ΔH* ^‡^ of **1-Me** by coalescence method using VT-^1^H-NMR spectra of **1-Me** (see the Supplementary Materials for details) (Leigh et al., 1998; Kawasaki et al., 1999; Furusho et al., 2004; Kihara et al., 2007; Berná et al., 2012). The signals of the two *O*-benzylic protons (a and a’) gradually broadened and eventually splitted with a decrease in temperature, showing the coalescence temperature (*T*_c_) around 25°C in 400 MHz-^1^H NMR (Figures 3B,C, and Supplementary Figures S36–S39). In 500 MHz-^1^H NMR, *T*_c_ of *O*-benzylic protons (a and a’) of **1-Me** appeared around 30°C (Supplementary Figures S38, S39, Supplementary Table S1). The estimated rate constants were also provided in the Supplementary Table S1. The obtained thermodynamic parameters are as follows: *E* = 17 kJ/mol, *ΔG* ^‡^ = 56 kJ/mol (at 25°C), *ΔH* ^‡^ = 17 kJ/mol, and *ΔS* ^‡^ = –141 J/mol⋅K (see the Supplementary Material for details, Supplementary Table S1, Supplementary Figures S40, S41). The small *ΔH* ^‡^ and big negative *ΔS* ^‡^ simply indicated that the mechanostereoinversion is an entropy-driven process (see the Supplementary Material for details). On the other hand, no *T*_c_ was confirmed until 140°C for all other rotaxanes, ***rac*-1-Et**, ***rac*-1-Ac** and ***rac*-1-Bz** in VT-NMR in DMSO-*d*_6_, being unsuccessful determination of thermodynamic parameters by VT-NMR.

### Enantiomer Separation of 1-Rs

**FIGURE 4:**
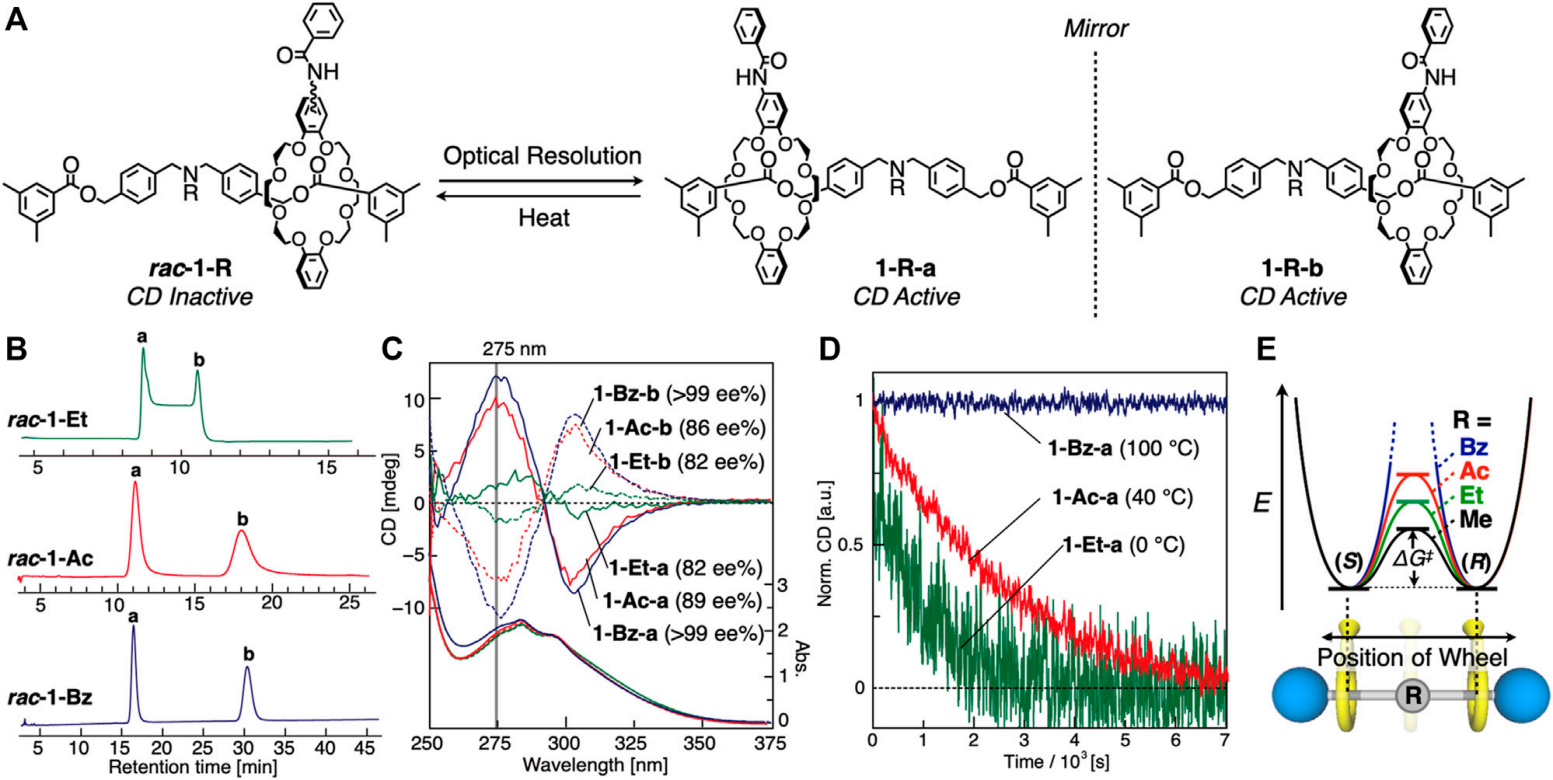
**(A)** Enantiomer separations of ***rac*-1-R** and racemization of **1-R-a** and **1-R-b**. **(B)** Chiral HPLC profiles (CHIRALPAK IA^®^) of ***rac*-1-Et** (at 333 K), ***rac*-1-Ac** (at 283 K), and ***rac*-1-Bz** (at 283 K). The determination of the rate constant of mechanostereoinversion by dynamic HPLC method was shown in Supplementary Figures S43–S45 and Supplementary Table S2. Absolute structures of mechanically chiral rotaxanes could not be determined. **(C)** CD and UV spectra of **1-Et-a**/**b**, **1-Ac-a**/**b** and **1-Bz-a**/**b** (0.1 mM, CHCl_3_, 263 K). **(D)** CD decay profiles of **1-Et-a** (at 273 K), **1-Ac-a** (at 313 K) and **1-Bz-a** (at 373 K) (0.1 mM, CHCl_3_, 275 nm). **(E)** Energy diagrams of **1-R**s.

The enantiomer separations of ***rac*-1-Et**, ***rac*-1-Ac**, and ***rac*-1-Bz** were performed with a chiral HPLC (Figure 4A). Two enantiomers were successfully separated in each rotaxane at 10°C (Figure 4B, and Supplementary Figures S33–S35): **1-Et-a**, **1-Et-b**, **1-Ac-a**, **1-Ac-b**, **1-Bz-a** and **1-Bz-b** (-a means first eluted fraction and -b means second eluted fraction). However, the enantiomer separation of ***rac*-1-Et** was not successful at 40°C (Figure 4B). The HPLC profile is typical for the compounds that undergo racemization in chiral stationary phase (Trapp 2006) (Figure 4B, Supplementary Figure S43). This observation indicates that ***rac*-1-Et** has lower stereoinversion energy than ***rac*-1-Ac** and ***rac*-1-Bz** because ethyl group is smaller than acetyl and benzoyl group. In fact, the enantiomer separation of **1-Et** performed at 10°C resulted in successful enantiomer separation (Supplementary Figure S33). We then confirmed that they are indeed enantiomer each other by mirror imaged CD spectra (Figure 4C), and determined that the optical purities were 82 ee% (**1-Et-a**), 82 ee% (**1-Et-b**), 89 ee% (**1-Ac-a**), 86 ee% (**1-Ac-b**), >99 ee% (**1-Bz-a**), and >99 ee% (**1-Bz-b**) by chiral HPLC (Supplementary Figures S33–S35). Weaker CD intensities of 1-Et-a and 1-Et-b than those of 1-Ac-a and 1-Ac-b, and 1-Bz-a and 1-Bz-b would be their original property, not only due to its low ee%. Clearly from the inspections of Figure 4C, [2]rotaxanes consisting of an unsymmetrical wheel component and a symmetrical axle component can generate co-conformationally mechanically planer chirality when the relative component arrangement was off-centered, and the two enantiomers can be interconverted *via* translational motion by overcoming the central steric barrier on the axle (Figure 1B).

### Racemization Behaviors of Optically Active 1-Rs

Then we investigated the racemization behaviors of these rotaxane enantiomers by tracing CD decay (Figure 4D), as Saito and co-workers have performed (Mochizuki et al., 2017). The CD intensities of **1-Et** and **1-Ac** decreased with time, yielding the thermodynamic parameters of **1-Et** and **1-Ac** (see the Supplementary Material for details, Supplementary Figures S42–S49, Supplementary Tables S2–S5) as summarized in Table 1. Here, the racemization of **1-Ac** was unexpected according to our previous report (Kihara et al., 2007) on the of steric barrier on the shuttling of various rotaxanes having DB24C8 wheel. We did not detect the shuttling behavior of the rotaxanes with *N*-acetylated axle by ^1^H NMR using peak coalescence method. The racemization results of dynamically chiral rotaxanes made possible the evaluation of the shuttling behavior of the rotaxanes with high steric barrier which cannot be detected by NMR.

**TABLE 1:** Thermodynamic parameters in mechanostereoinversion of 1-Rs.

	1-Me	1-Et	1-Ac	1-Bz
*E* (kJ/mol)	17	42	73	—
*ΔG* ^‡^ (kJ/mol)	54^a^	88^b^	99^c^	—
*ΔH* ^‡^ (kJ/mol)	15	46	71	—
*ΔS* ^‡^ (J/mol⋅K)	–141	–61	–119	—

^a^ By ^1^H NMR at 298 K in CDCl_3_.

^b^ By CD decay trace at 283 K in CHCl_3_. Thermodynamic parameters of **1-Et** estimated with the data obtained from dynamic HPLC are shown in Supplementary Tables S2, S3.

^c^ By CD decay trace at 313 K in CHCl_3_.

As expected, *E*, *ΔG* ^‡^, *ΔH* ^‡^ of **1-Ac** for racemization are larger than those of **1-Et** (Table 1) because acetyl group of **1-Ac** is bulkier than ethyl group of **1-Et**. On the other hand, CD intensity of **1-Bz** never decreased even at 100°C in DMSO (Figure 4D), suggesting that *N*-Bz group is bulky enough to prevent completely the wheel overcoming it. It is clear that the racemization energy of such [2]rotaxanes is controlled by the bulkiness of the central substituent group of the axle component (Figure 4E). According to the increase in bulkiness of the substituent, the *E*, *ΔG* ^‡^ and *ΔH* ^‡^ values increase, but the *ΔS* ^‡^ does not (Table 1). The tendency of the change in *ΔS* ^‡^ value depending on the substituent groups on *N*-atom is unclear at present.

### Chiral-Prochiral Interconversion

**FIGURE 5:**
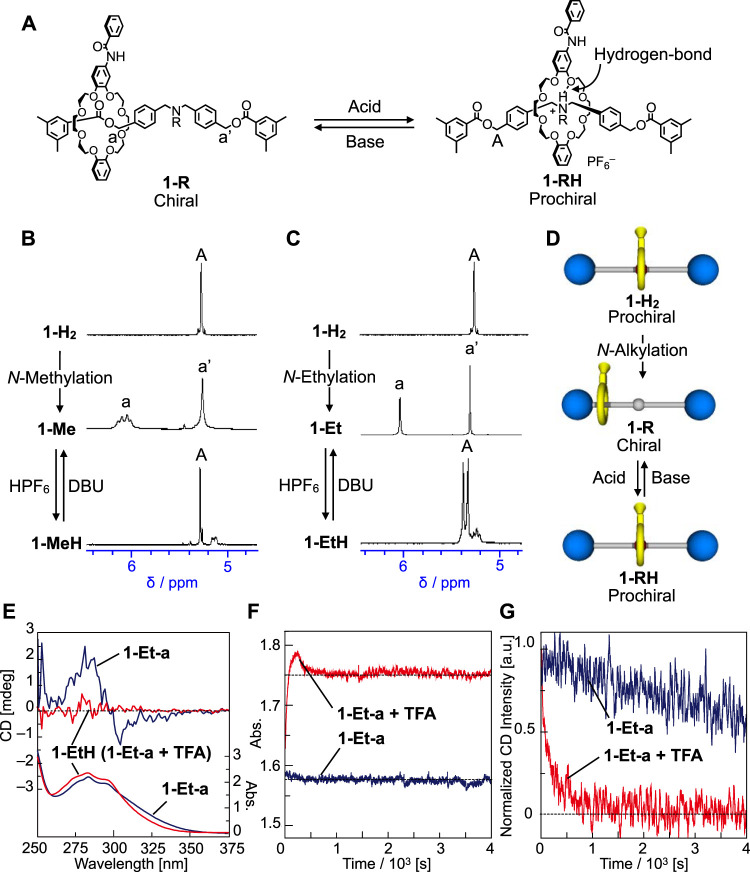
**(A)** Protonation and deprotonation of the nitrogen moiety of **1-R** and **1-RH**. Absolute structures of mechanically chiral rotaxanes could not be determined. **(B)** ^1^H NMR spectra (400 MHz, CDCl_3_) of **1-H**_**2**_ (298 K), **1-Me** (213 K) and **1-MeH** (298 K). **(C)** ^1^H NMR spectra (400 MHz) of **1-H**_**2**_ (CDCl_3_, 298 K), **1-Et** (DMSO-*d*_6_, 413 K) and **1-EtH** (CDCl_3_, 298 K). **(D)** Chiral structural changes of **1-R**s and **1-RH**s. **(E)** CD and UV spectra of **1-Et-a** and **1-EtH** (0.1 mM, CHCl_3_, 263 K). **(F)** UV absorption intensity profiles at 275 nm and **(G)** CD decay profiles at 275 nm of **1-Et-a** during the protonation by TFA (red profiles, 0.1 mM, CHCl_3_, 263 K). As a reference, the UV and CD profiles of **1-Et-a** without addition of TFA are shown [blue profiles, in **(F)** and **(G)**]. Since simple racemization of **1-Et-a** occurs without addition of TFA, UV spectrum did not change [**(F)**, blue] and CD intensity decreased much slower than that with addition of TFA [**(G)**, blue].

Next, we tried the chiral-prochiral interconversion of rotaxane *via* switching technique. When *tert*-amine group of **1-Me** and **1-Et** is protonated to generate *tert*-ammonium group, the crown ether moves and localizes onto the central *tert*-ammonium group due to the hydrogen-bond between the crown ether and *tert*-ammonium proton (Figures 5A–D) (Nakazono et al., 2008; Suzuki et al., 2010; Ishiwari et al., 2011; Suzuki et al., 2012). As a result, the rotaxane will become a prochiral again (Figures 1B, 2, 5D). We treated **1-Me** and **1-Et** with HPF_6_ to generate **1-MeH** and **1-EtH** (Figure 5A), then investigated their NMR spectra (Figures 5B,C, see the Supplementary Material for details). The NMR signals of the axle component of **1-MeH** are completely symmetry (Figure 5B), and the chemical shift of *O*-benzyl proton (a or A) reverted to the almost same position as prochiral **1-H**_**2**_, meaning that the wheel component moved back and localized at the center of axle component. Therefore **1-MeH** became prochiral and lost its chirality (Figure 5D, see the Supplementary Material for details). Furthermore, prochiral **1-RH** can be easily converted to chiral **1-R** by base treatment such as 1,8-diazabicyclo [5.4.0]undec-7-ene (DBU) (Figures 5A–D). Thus, we succeeded in chiral-prochiral interconversion of rotaxane by protonation and neutralization for central amine group (Figure 2A). With respect to **1-Et**, the protonated **1-EtH** showed more complicated NMR spectra than **1-MeH**. First of all, the ^1^H signals of *O*-benzyl protons of **1-EtH** appeared non-equivalently, suggesting that the wheel component is located slightly off-center of the axis (Supplementary Figure S50), which causes co-conformationally mechanical planar chirality at the NMR timescale. In addition, the proton is strongly bonded to *N*-atom and the dissociation from *N*-atom is slower than the NMR timescale due to surrounding hydrogen-bonding from the crown ether, which also causes co-conformationally mechanical point chirality on *N*-atom at the NMR timescale. Thus, **1-EtH** can be regarded as a diastereomeric species at the NMR timescale, which makes the NMR spectra of **1-EtH** complicated. If the time scale of the isomerism of the chiralities of **1-EtH** is faster than the experimental time scale, **1-EtH** can be virtually regarded as prochiral structure (Figure 5D and Supplementary Figure S50). That can be discussed from CD trace experiment at the next paragraph. However, at this stage, analogous to the case of **1-MeH**, the signals of *O*-benzyl protons (A) of **1-EtH** appeared at similar chemical shift to that of **1-H**_**2**_, indicating that the wheel component located similar position to that of **1-H**_**2**_, *i.e.*, near the central ammonium group.

Then the protonation experiment was carried out to the optically active **1-Et-a** (Figure 5A). We traced the CD and UV absorption intensities of **1-Et-a** after adding 1.5 eq. of trifluoroacetic acid (TFA) at –10°C (Figures 5E–G). Upon addition of TFA, the CD intensity decreased immediately and disappeared by 1000 s (Figure 5G, red). Although, UV absorption changed in a complicated way (Figure 5F, red), and the changes of UV and CD spectra started and finished almost simultaneously. Which means that the protonation step seems the rate-controlling step for the racemization or the chirality loss. If the isomerism of the chiralities of **1-EtH** was slower than the experimental time scale, the CD activity should be maintained after protonation (*i.e.*, change in UV absorption) finished, but this was not the case in the experimental results. These observations suggest that **1-EtH** is observed as a diastereomeric species at the NMR timescale, but can be regarded as prochiral at an ordinary experimental timescale (Supplementary Figure S50). Generated **1-EtH** can be also converted to **1-Et** by neutralization *via* DBU. These results indicate that we can control the racemization rate and energy diagram of dynamically chiral [2]rotaxane by chiral-prochiral interconversion *via* protonation and neutralization (Figures 1B, 2, 5).

## Conclusion

In conclusion, a series of [2]rotaxanes consisting of an unsymmetrical wheel component with *C*_s_ symmetry and a symmetrical axle with *C*_2v_ symmetry were systematically synthesized and characterized: the rotaxanes showed the dynamic mechanical chirality, and the racemization energy of the isolated single enantiomers was managed by the control of the bulkiness of the central substituents of the axle component. We also found that the results of the racemization of the dynamically chiral rotaxane can clarify the shuttling behavior of the rotaxanes with high steric barrier on the shuttling which cannot be detected by NMR. Furthermore, we demonstrated the chiral-prochiral interconversion by positional switching technique of rotaxane. The present systematic studies will provide new insight into mechanically chiral interlocked compounds as well as utility as dynamic chiral source using switching technique of rotaxanes have been reported toward molecular device systems (Kay et al., 2007; Brain et al., 2012). Utilization of rotaxane with dynamic mechanical chiral as chiral sources and asymmetric synthesis of optically active [2]rotaxane from prochiral **1-RH** are under way.

## Experimental section

### Materials and methods

Commercially available materials and solvents, including NaBH(AcO)_3_ (TCI), paraformaldehyde (Nakarai Tesque, Ltd.), hexafluorophosphoric acid (Aldrich), 1,8-diazabicyclo [5,4,0]undec-7-ene (Aldrich), *N*-methylpyrrolidone (NMP, Wako Pure Chemical Industries, Ltd.), and tributylphosphine (TCI) were used without further purification. Column chromatography was performed using Wakogel C-400HG (SiO_2_, Wako Pure Chemical Industries Ltd.) and Merck Aluminum Oxide 90 (Al_2_O_3_) standardized. 3,5-Dimethylbenzoic anhydride **2**) (Tachibana et al., 2006), crown ethers **3** (Makita et al., 2007) and *sec*-ammonium salt **4** (Hirose et al., 2007) were prepared according to the literature.

^1^H (400 MHz) and ^13^C (100 MHz) NMR spectra were recorded on a JEOL AL-400 spectrometer using CDCl_3_ or DMSO-*d*_6_ as the solvent, and tetramethylsilane or residual solvents as the internal standard. ^1^H (500 MHz) NMR spectra were recorded on a Bruker biospin AVANCE III HD500 spectrometer using CDCl_3_ as the solvent, and tetramethylsilane as the internal standard. Samples were purified by repeated preparative gel permeation chromatography (GPC) on a JAI Co., Ltd. LC-9204 system (JAIGEL-1H-40) with CHCl_3_ as the eluent. IR spectra were recorded on a JASCO FT/IR-230 spectrometer. Melting points were measured with a Stuart Scientific SMP3 (Bibby Scientific). UV-vis spectra were taken on a JASCO V-550 UV-vis spectrophotometer. Enantiomer separations and the determination of the enantiomeric excess values were carried out by chiral HPLC on a JASCO HSS-1500 System equipped with CHIRALPAK IA^®^ (25 cm × 2.0 cmφ for the separation, 25 cm × 4.6 mmφ for the analysis) isocratically eluted with *n*-hexane/CHCl_3_/Et_2_NH = 1/1/0.005 for **1-Et** and CHCl_3_/*n*-hexane (1/1, v/v) for **1-Ac** and **1-Bz** at flow rates of 3.0 ml/min at 283 K for separation and 1.0 ml/min at suggested temperatures for analysis. CD spectra were taken on a JASCO J-820 spectropolarimeter. High-resolution mass spectra (HR-MS) data were recorded by the National University Corporation, Tokyo Institute of Technology, Center for Advanced Materials Analysis, on request.

### Synthesis

**1-H**_**2**_. To a solution of benzamide substituted DB24C8 **3** (567 mg, 1.00 mmol) and *sec*-ammonium salt **4** (403 mg, 1.0 mmol) in CHCl_3_ (10 ml) was added PBu_3_ (14 μL, 0.05 mmol) and 3,5-dimethylbenzoicacid anhydride **2**) (900 mg, 3.0 mmol) at room temperature, and stirred for 12 h. The reaction solution was poured into *n*-hexane (70 ml) and the precipitates were collected by decantation and purified by SiO_2_ column chromatography (CHCl_3_/EtOAc = 1/1) and recycle preparative GPC (CHCl_3_) to give rotaxane **1-H**_**2**_ (1.00 g, 0.81 mmol, 81%) as colorless foam: mp 132.1–133.9°C. ^1^H NMR (400 MHz, CDCl_3_, 298 K) *δ* 8.94 (s, 1H), 8.00 (dd, 2H, *J* = 7.6, 1.6 Hz), 7.65 (s, 4H), 7.57 (br, 2H), 7.50–7.45 (m, 3H), 7.41 (dd, 1H, *J* = 8.6, 1.8 Hz), 7.30 (d, 4H, J = 8.6 Hz), 7.28 (d, 4H, *J* = 8.6 Hz), 7.19 (s, 2H), 6.87–6.83 (m, 2H), 6.77–6.72 (m, 2H), 6.67 (d, 1H, *J* = 8.6 Hz), 5.29 (d, 2H, *J* = 14.2 Hz), 5.25 (d, 2H, *J* = 14.2), 4.63–4.58 (m, 4H), 4.14–4.13 (m, 2H), 4.11–4.10 (m, 2H), 4.06–4.03 (m, 4H), 3.79–3.78 (m, 4H), 3.73–3.71 (m, 6H), 3.47–3.41 (m, 8H), 2.34 (s, 12H) ppm. ^13^C NMR (100 MHz, CDCl_3_, 298 K) *δ* 166.5, 165.6, 147.2, 146.9, 143.6, 138.0, 137.4, 134.8, 134.3, 132.8, 131.5, 131.2, 129.6, 129.2, 128.4, 127.8, 127.2, 121.5, 113.1, 112.5, 112.3, 106.2, 70.5, 70.3, 70.0, 68.0, 67.9, 65.5, 52.1, 21.0 ppm. FT-IR (KBr) *ν* 3416, 3145, 2922, 1716, 1668, 1607, 1514, 1454, 1407, 1384, 1354, 1308, 1254, 1215, 1114, 1058, 1011, 985, 954, 843, 768, 745, 710, 602, 558, 471 cm^−1^. HRMS (ESI) [M–PF_6_]^+^ calcd’ for C_65_H_73_N_2_O_13_: 1089.5107, found 1089.5176.

***rac*-1-Me.** A solution of rotaxane **1-H**_**2**_ (200 mg, 162 µmol), paraformaldehyde (97 mg, 3.24 mmol), NaBH(AcO)_3_ (170 mg, 0.81 mmol), and triethylamine (1 ml) in NMP (4 ml) was stirred for 8 h at room temperature. The reaction mixture was then poured into water (300 ml) and the precipitate was collected by filtration, dissolved in EtOAc, washed with H_2_O, sat. NaHCO_3_ aq. and brine, dried over MgSO_4_, and concentrated *in vacuo*. The residue was purified by flash Al_2_O_3_ column chromatography (EtOAc) to give rotaxane ***rac-*1-Me** (166 mg, 151 μmol, 93%) as colorless foam: mp 112.8–114.1°C. ^1^H NMR (400 MHz, CDCl_3_, 233 K) *δ* (8.87, s, 1H), 8.43 (d, 2H, *J* = 6.8 Hz), 8.20 (s, 2H), 7.95 (d, 2H, *J* = 6.8 Hz), 7.70 (s, 2H), 7.51–7.48 (m, 3H), 7.42–7.39 (m, 2H), 7.31–7.27 (m, 2H), 7.30–7.17 (m, 7H), 6.95–6.92 (m, 4H), 6.75 (d, 1H, *J* = 8.2 Hz), 6.08 (d, 1H, *J* = 16.8 Hz), 6.04 (d, 1H, *J* = 16.8), 5.27 (s, 2H), 4.09 (br, 8H), 3.64–3.63 (m, 8H), 3.46 (s, 2H), 3.45 (s, 2H), 3.12 (br, 4H), 2.58–2.48 (m, 4H), 2.36 (s, 6H), 2.21 (s, 6H), 2.17 (s, 3H) ppm. ^1^H NMR (400 MHz, CDCl_3_, 333 K) *δ* 7.86 (s, 4H), 7.85 (dd, 2H, *J* = 7.4, 1.5 Hz), 7.69 (br, 4H), 7.52–7.43 (m, 3H), 7.34 (d, 1H, *J* = 2.0 Hz), 7.19 (d, 4H, *J* = 7.7 Hz), 7.09 (s, 2H), 6.95 (dd, 1H, *J* = 8.8, 2.0 Hz), 6.89–6.77 (m, 4H), 6.75 (d, 1H, *J* = 8.6 Hz), 5.64 (s, 4H), 4.11–4.08 (m, 4H), 3.68–3.64 (m, 8H), 3,42 (s, 4H), 3.14 (br, 8H), 2.26 (s, 12H), 2.11 (s, 3H) ppm. ^13^C NMR (100 MHz, CDCl_3_, 333 K) *δ* 167.1, 165.5, 148.7, 145.8, 137.7, 135.7, 135.4, 134.2, 134.1, 131.4, 130.8, 128.7, 128.5, 127.9, 127.8, 127.1, 127.0, 120.5, 112.0, 111.8, 111.5, 105.8, 105.7, 69.6, 68.4, 68.2, 68.0, 66.9, 61.4, 42.2, 42.1, 21.0, 20.9, 20.8, 20.7 ppm. FT-IR (KBr) *ν* 3791, 3430, 2923, 2876, 1715, 1665, 1608, 1513, 1453, 1423, 1381, 1309, 1253, 1218, 1126, 1055, 955, 870, 801, 769, 743, 709, 469 cm^−1^. HRMS (FAB) [M + H]^+^ calcd’ for C_66_H_75_N_2_O_13_: 1103.5269, found 1103.5271.

***rac*-1-MeH.** A solution of ***rac*-1-Me** (100 mg, 90.7 μmol) in CH_2_Cl_2_ (50 ml) was washed with 10% HPF_6_ aq (*Caution! Hazardous!* 50 ml x 3). Then the organic layer was washed with brine, dried over MgSO_4_, and concentrated *in vacuo*. The residue was purified by flash SiO_2_ column chromatography (CHCl_3_/MeOH = 95/5) to give rotaxane ***rac-*1-MeH** (111 mg, 88.9 µmol, 98%) as colorless foam: mp 125.5–131.1°C. ^1^H NMR (400 MHz, CDCl_3_, 333 K) *δ* 8.32 (s, 1H), 7.96 (d, 2H, *J* = 7.9 Hz), 7.65 (s, 4H), 7.58–7.55 (m, 2H), 7.46–7.42 (m, 3H), 7.37–7.33 (m, 2H), 7.29 (d, 4H, *J* = 7.9 Hz), 7.18–7.17 (m, 2H), 6.86–6.84 (m, 2H), 6.76–6.71 (m, 3H), 5.21–5.19 (m, 4H), 5.11–5.09 (m, 2H), 4.46–4.41 (m, 2H), 4.21–4.07 (m, 8H), 3.68–3.66 (m, 8H), 3.56–3.50 (m, 8H), 2.96 (m, 3H), 2.34–2.33 (m, 12H) ppm. ^13^C NMR (100 MHz, CDCl_3_, 298 K) *δ* 166.5, 165.7, 147.3, 147.2, 147.1, 147.0, 143.9, 143.8, 138.1, 137.9, 134.7, 132.7, 131.8, 131.5, 130.1, 130.0, 128.6, 128.0, 127.4, 127.3, 121.5, 121.4, 113.2, 113.1, 112.1, 112.0, 111.9, 111.8, 111.7, 106.3, 106.1, 71.8, 71.7, 71.6, 71.5, 70.7, 70.6, 70.5, 70.4, 68.7, 68.4, 68.3, 68.1, 65.7, 60.6, 39.6, 21.0 ppm. FT-IR (KBr) *ν* 3417, 3063, 2922, 2878, 1716, 1667, 1607, 1513, 1455, 1309, 1251, 1214, 1116, 1058, 955, 843, 768, 746, 710, 557 cm^−1^.

***rac*-1-Et.** Under Ar atmosphere, a solution of rotaxane **1-H**_**2**_ (200 mg, 162 µmol), NaBH(AcO)_3_ (170 mg, 0.81 mmol), and triethylamine (1 ml) in NMP (4 ml) was stirred for 8 h at 70°C. After cooling to room temperature, the reaction mixture was then poured into water (300 ml) and the precipitate was collected by filtration, dissolved in EtOAc, washed with H_2_O, sat. NaHCO_3_ aq. and brine, dried over MgSO_4_, and concentrated *in vacuo.* The residue was purified by flash Al_2_O_3_ column chromatography (EtOAc) to give rotaxane ***rac-*1-Et** (157 mg, 141 μmol, 87%) as colorless foam: mp 109.2–111.2°C. ^1^H NMR (400 MHz, CDCl_3_, 333 K) *δ* (8.23, s, 1H), 8.21 (d, 2H, *J* = 8.2 Hz), 8.13 (s, 2H), 7.90 (d, 2H, *J* = 7.3 Hz), 7.69 (s, 2H), 7.50 (t, 1H, *J* = 7.3 Hz), 7.44 (dd, 2H, *J* = 7.3, 7.3 Hz), 7.41 (s, 1H), 7.32 (d, 2H, *J* = 8.5 Hz), 7.30 (d, 2H, *J* = 8.5 Hz), 7.18 (s, 2H), 7.17 (d, 2H, *J* = 7.8 Hz), 7.09 (s, 2H), 7.08 (d, 1H, J = 8.8 Hz), 6.92–6.88 (m, 2H), 6.86–6.82 (m, 2H), 6.75 (d, 1H, *J* = 8.8 Hz), 6.03 (s, 2H), 5.30 (s, 2H), 4.05 (br, 8H), 3.66–3.63 (m, 8H), 3.49 (s, 2H), 3.47 (s, 2H), 3.22–3.21 (m, 4H), 2.84–2.82 (m, 4H), 2.43 (q, 2H, *J* = 7.1 Hz), 2.34 (s, 6H), 2.20 (s, 6H), 1.01 (t, 3H, *J* = 7.1 Hz) ppm. ^13^C NMR (100 MHz, CDCl_3_, 298 K) *δ* 167.0, 166.9, 165.5, 148.4, 148.3, 145.4, 140.6, 138.0, 137.6, 136.4, 136.1, 135.0, 134.6, 134.1, 134.0, 131.5, 131.3, 130.7, 129.9, 128.8, 128.6, 128.4, 128.1, 127.9, 127.3, 127.0, 120.4, 111.9, 111.3, 111.1, 105.3, 69.4, 69.3, 69.0, 68.1, 68.0, 67.8, 66.5, 57.2, 56.8, 46.8, 29.6, 21.1, 20.7, 12.0 ppm. FT-IR (KBr) *ν* 2962, 2924, 2873, 1716, 1607, 1541, 1508, 1456, 1420, 1376, 1310, 1254, 1219, 1127, 1053, 869, 803, 769, 744, 711, 606 cm^−1^. HRMS (FAB) [M + H]^+^ calcd’ for C_67_H_77_N_2_O_13_: 1117.5426, found 1117.5435.

***rac-*1-EtH.** The solution of ***rac*-1-Et** (50 mg, 44.8 μmol) in CH_2_Cl_2_ (50 ml) was washed with 10% HPF_6_ aq. (*Caution! Hazardous!* 50 ml x 3). Then the organic layer was washed with brine, dried over MgSO_4_, and concentrated *in vacuo*. The residue was purified by flash SiO_2_ column chromatography (CHCl_3_/MeOH = 95/5) to give rotaxane ***rac-*1-EtH** (55.4 mg, 43.9 µmol, 98%) as colorless foam: mp 99.7–102.2°C. ^1^H NMR (400 MHz, CDCl_3_, 333 K) *δ* (8.54, s, 0.5H), 8.47 (s, 0.5H), 8.20–8.18 (m, 2H), 8.00–7.98 (m, 2H), 7.68 (s, 2H), 7.65 (s, 2H), 7.50–7.43 (m, 6H), 7.33 (dd, 1H, *J* = 7.9, 3.2 Hz), 7.20 (s, 1H), 7.20 (s, 1H), 7.20–7.11 (m, 2H), 6.89–6.92 (m, 6H), 5.37 (s, 2H), 5.32 (s, 2H), 5.33–5.23 (m, 2H), 4.63–4.40 (m, 2H), 4.36–3.87 (m, 8.5H), 3.78–3.49 (m, 8.5H), 3.43–3.26 (m, 5H), 3.05–2.84 (m, 4H), 2.34 (s, 12H), 1.08–1.04 (m, 3H) ppm. ^13^C NMR (100 MHz, CDCl_3_, 298 K) *δ* 166.7, 166.5, 165.9, 147.3, 147.2, 147.1, 143.6, 138.2, 138.2, 138.1, 137.2, 137.2, 135.1, 134.9, 134.3, 133.8, 133.6, 133.3, 133.1, 131.7, 131.3, 129.8, 129.6, 128.6, 128.4, 128.3, 127.6, 127.4, 127.3, 121.8, 121.7, 121.6, 121.3, 113.3, 112.6, 112.5, 112.2, 112.0, 111.8, 107.4, 107.3, 106.3, 71.4, 71.2, 71.1, 70.9, 70.5, 70.2, 70.0, 69.8, 69.8, 69.4, 69.1, 68.9, 68.8, 68.7, 68.5, 68.2, 66.1, 65.6, 59.0, 54.3, 54.2, 45.5, 45.2, 21.2, 21.1, 14.1, 9.4, 8.9 ppm. FT-IR (KBr) *ν* 3416, 3061, 2957, 2925, 1717, 1666, 1607, 1513, 1453, 1309, 1261, 1214, 1113, 1056, 954, 844, 768, 746, 710, 558 cm^−1^.

***rac-*1-Ac.** Under Ar atmosphere, a solution of rotaxane **1-H**_**2**_ (200 mg, 162 µmol), triethylamine (101 μL, 648 µmol), and acetic anhydride (31 μL, 324 µmol) in dry DMF (1 ml) was stirred for 8 h at room temperature. The reaction mixture was then poured into water (300 ml) and the precipitate was collected by filtration, dissolved in EtOAc, washed with H_2_O, sat. NaHCO_3_ aq. and brine, dried over MgSO_4_, and concentrated *in vacuo*. The residue was purified by flash SiO_2_ column chromatography (CHCl_3_/MeOH = 95/5) to give rotaxane ***rac-*1-Ac** (137 mg, 121 μmol, 75%) as colorless foam. mp 132.6–134.1°C. ^1^H NMR (400 MHz, DMSO-*d*_6_, 413 K) *δ* (9.60, s, 1H), 8.04 (d, 2H, *J* = 7.8 Hz), 7.96 (s, 2H), 7.93 (d, 2H, *J* = 7.8 Hz), 7.59 (s, 2H), 7.53–7.46 (m, 3H), 7.42 (s, 1H), 7.36 (d, *J* = 7.8 Hz), 7.29–7.26 (m, 1H), 7.26 (s, 1H), 7.16 (d, 2H, *J* = 7.8 Hz), 7.11 (s, 1H), 6.90–6.82 (m, 7H), 5.95 (s, 2H), 5.31 (s, 2H), 4.37 (s, 4H), 4.08–4.05 (m, 4H), 4.01–3.98 (m, 4H), 3.71–3.67 (m, 4H), 3.59–3.54 (m, 4H), 3.32–3.31 (m, 4H), 3.11–3.08 (m, 4H), 2.33 (s, 6H), 2.21 (s, 6H), 2.01 (s, 3H) ppm. ^13^C NMR (100 MHz, CDCl_3_, 333 K) *δ* 170.9, 170.8, 167.1, 167.0, 166.8, 165.6, 148.4, 148.3, 148.2, 145.5, 145.4, 138.1, 138.0, 137.8, 137.6, 137.5, 137.4, 136.7, 136.6, 135.4, 135.1, 134.9, 134.8, 134.7, 134.2, 134.1, 133.9, 133.1, 131.5, 131.2, 131.1, 130.7, 130.6, 129.8, 129.0, 128.8, 128.6, 128.5, 128.4, 128.3, 128.2, 127.3, 127.1, 126.8, 126.7, 125.6, 120.4, 120.3, 113.1, 112.7, 111.4, 111.1, 111.0, 106.3, 106.1, 69.6, 69.4, 69.2, 69.1, 67.8, 66.9, 66.6, 66.2, 66.1, 50.7, 49.8, 47.3, 46.9, 21.7, 21.1, 20.8 ppm. FT-IR (KBr) *ν* 2923, 2854, 1713, 1642, 1608, 1513, 1452, 1422, 1381, 1309, 1253, 1220, 1127, 1054, 1011, 769, 742, 709, 607 cm^−1^. HRMS (FAB) [M + H]^+^ calcd’ for C_67_H_75_N_2_O_14_: 1131.5218, found 1131.5164.

***rac*-1-Bz.** Under Ar atmosphere, a solution of rotaxane **1-H**_**2**_ (200 mg, 162 µmol), triethylamine (101 μL, 648 µmol), and benzoyl chloride (37 μL, 324 µmol) in dry DMF (1 ml) was stirred for 8 h at room temperature. The reaction mixture was then poured into water (300 ml) and the precipitate was collected by filtration, dissolved in EtOAc, washed with H_2_O, sat. NaHCO_3_ aq. and brine, dried over MgSO_4_, and concentrated *in vacuo*. The residue was purified by flash SiO_2_ column chromatography (CHCl_3_/MeOH = 95/5) to give rotaxane ***rac-*1-Bz** (126 mg, 105 μmol, 65%) as colorless foam. mp 126.3–128.2°C. ^1^H NMR (400 MHz, DMSO-*d*_6_, 413 K) *δ* (9.57, s, 1H), 8.10 (d, 2H, J = 8.1 Hz), 7.96 (s, 2H), 7.93 (d, 2H, J = 8.1 Hz), 7.60 (s, 2H), 7.54–7.45 (m, 3H), 7.43 (s, 1H), 7.38 (d, 2H, J = 7.2 Hz), 7.38–7.33 (m, 5H), 7.29–7.26 (m, 1H), 7.29 (dd, 1H, *J* = 8.7, 2.2 Hz), 7.26 (s, 1H), 7.16 (d, 2H, *J* = 7.2 Hz), 7.11 (s, 1H), 6.91–6.82 (m, 7H), 5.70 (s, 2H), 5.32 (s, 2H), 4.41 (s, 4H), 4.41 (s, 2H), 4.37 (s, 2H), 4.11–4.05 (m, 4H), 4.03–3.98 (m, 4H), 3.70–3.64 (m, 4H), 3.62–3.54 (m, 4H), 3.35–3.30 (m, 4H), 3.11–3.06 (m, 4H), 2.34 (s, 6H), 2.21 (s, 6H) ppm. ^13^C NMR (100 MHz, CDCl_3_, 298 K) *δ* 172.0, 167.2, 166.7, 165.5, 148.6, 148.6, 145.8, 138.0, 137.6, 137.2, 136.5, 135.5, 135.1, 134.6, 134.0, 131.4, 131.4, 131.1, 130.2, 129.4, 129.1, 128.5, 128.4, 128.3, 127.4, 127.1, 126.7, 126.0, 120.5, 113.0, 111.6, 111.4, 106.5, 69.7, 69.6, 69.5, 69.5, 68.1, 68.0, 66.2, 51.5, 46.4, 31.5, 21.0, 20.7 ppm. FT-IR (KBr) *ν* 3060, 2921, 2876, 1715, 1666, 1632, 1608, 1580, 1513, 1453, 1416, 1382, 1309, 1253, 1220, 1126, 1054, 1005, 952, 869, 846, 769, 743, 705, 681, 603, 558, 479, 408 cm^−1^. HRMS (FAB) [M + Na]^+^ calcd’ for C_72_H_76_N_2_O_14_Na: 1215.5194, found 1215.5181.

### Detailed Protocols of optical resolution of 1-Et

Enantiomer separation of **1-Et** was carried out using chiral HPLC at low temperature (10°C), and eluted fractions were collected to flasks cooled in ice bath with NaCl. The collected fractions were quickly concentrated using rotary evaporator in ice bath with NaCl, and then high vacuum pomp. The resultant optically active **1-Et** were immediately used for next experiments (CD measurements, chiral HPLC analysis, and protonation experiments), or stored at refrigerator at –40 °C to prevent racemization.

### Detailed Protocols protonation experiment for optically active 1-Et-a

The CHCl_3_ solution of optically active **1-Et-a (**0.1 mM) was quickly prepared using freshly prepared optically active **1-Et-a** in one dilution. Then, 3.5 ml of thus prepared 0.1 mM CHCl_3_ solution of optically active **1-Et-a** was quickly transferred to a 1.0 cm × 1.0 cm quartz cell cooled at –10°C with a Peltier cooling system equipped in CD measurement instrument, and measured first CD spectra at –10°C (Figure 5E, blue). And then, 5.0 µL of CHCl_3_ solution of TFA (0.105 mM) was added to the quartz cell, and the CD and UV changes were tracked at –10°C (Figures 5F,G, red). CD spectrum was again measured more than 4000 s passed after the addition of TFA (Figure 5E, red).

## Data Availability

The original contributions presented in the study are included in the article/Supplementary Meterial, further inquiries can be directed to the corresponding authors.
