# Supplementary material for: Rotaxanes with dynamic mechanical chirality: Systematic studies on synthesis, enantiomer separation, racemization, and chiral-prochiral interconversion
Source: Front Chem. 2022 Oct 28;10:1025977. doi: 10.3389/fchem.2022.1025977 (PMC9650364; doi:10.3389/fchem.2022.1025977)
Supplement: Supplementary file 1 [file DataSheet1.PDF]

Supplementary Material for

Rotaxanes with Dynamic Mechanical Chirality: Systematic Studies on Synthesis, Optical Resolution, Racemization, and Chiral-Prochiral Interconversion

Fumitaka Ishiwari[1,†,*], **Toshikazu Takata**[1,2,3,*]

[1] Department of Chemical Science and Engineering, Tokyo Institute of Technology, Ookayama, Meguro-ku, Tokyo 152-8552, Japan

[2] School of Materials and Chemical Technology, Tokyo Institute of Technology, Nagatsuta-cho, Midori-ku, Yokohama 226-8503, Japan

[3] Graduate School of Advanced Science and Engineering, Hiroshima University, 1-4-1 Kagamiyama, Higashi-Hiroshima, Hiroshima, 739-8527, Japan

*** Correspondence:**
Fumitaka Ishiwari
ishiwari@chem.eng.osaka-u.ac.jp
Toshikazu Takata
takatats@hiroshima-u.ac.jp

[†] Present Address: Department of Applied Chemistry, Graduate School of Engineering, Osaka University, 2-1 Yamadaoka, Suita, Osaka 565-0871, Japan.

Table of Contents

1. ^{1}H and ^{13}C NMR Spectra, HH COSY Correlations (Supplementary Figures S1–S25) 1

2. FT-IR Spectra (Supplementary Figures S26 and S27) 16

3. High-Resolution Mass Spectra (Supplementary Figures S28–S32) 18

4. Chiral HPLC Profiles (Supplementary Figures S33–S35) 21

5. Analysis of Racemization Behaviors (Supplementary Figures S36–S49 and Tables S1–S5) 23

6. Schematic Illustration (Supplementary Figure S50) 39

7. References 39

1. ^{1}H and ^{13}C NMR Spectra, HH-COSY Correlations

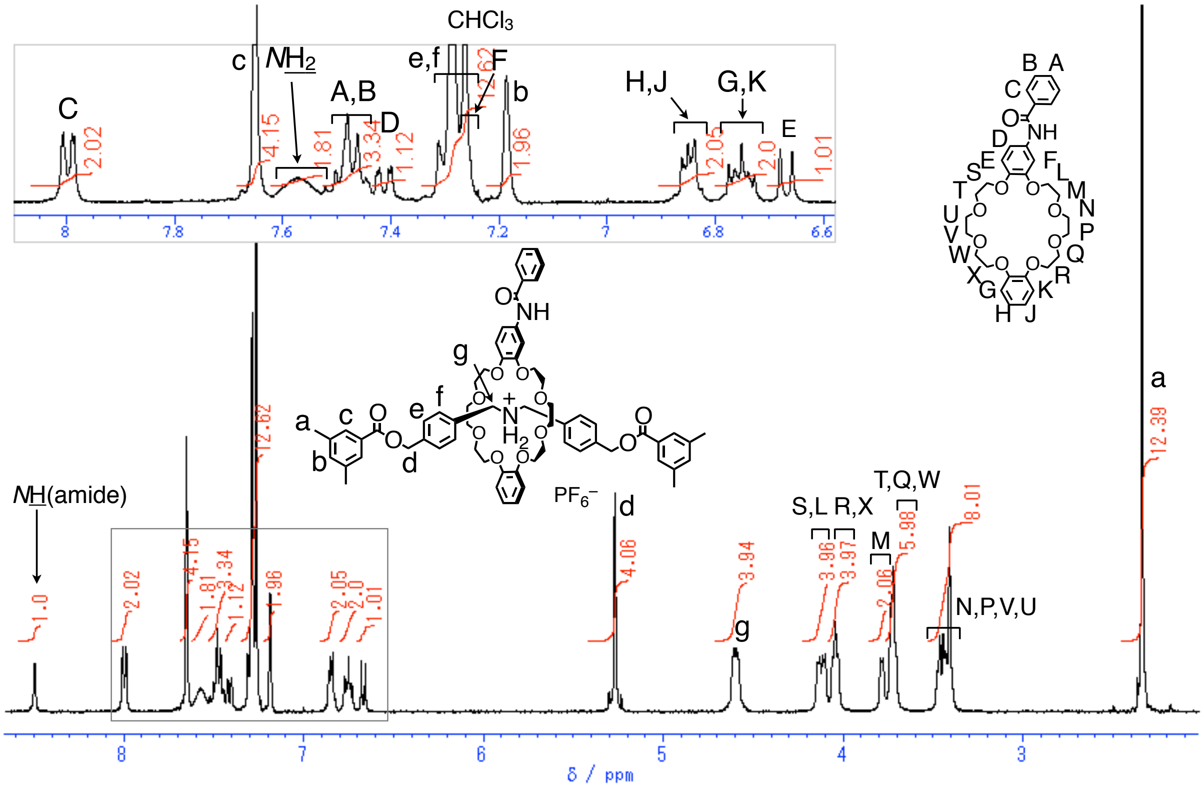

Supplementary Figure S1. ^{1}H NMR spectrum of **1-H$_2$** (400 MHz, $CDCl_3$, 298 K).

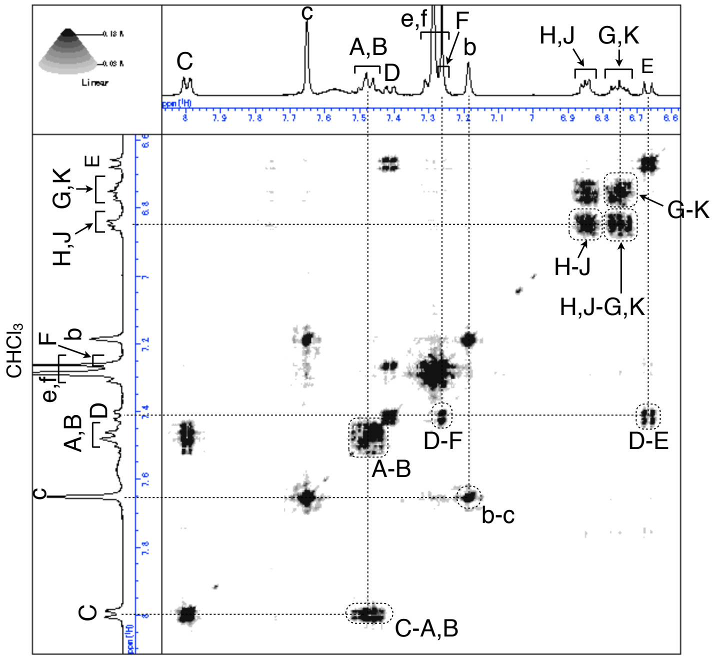

Supplementary Figure S2. Partial HH-COSY correlations of **1-H$_2$** (400 MHz, $CDCl_3$, 298 K).

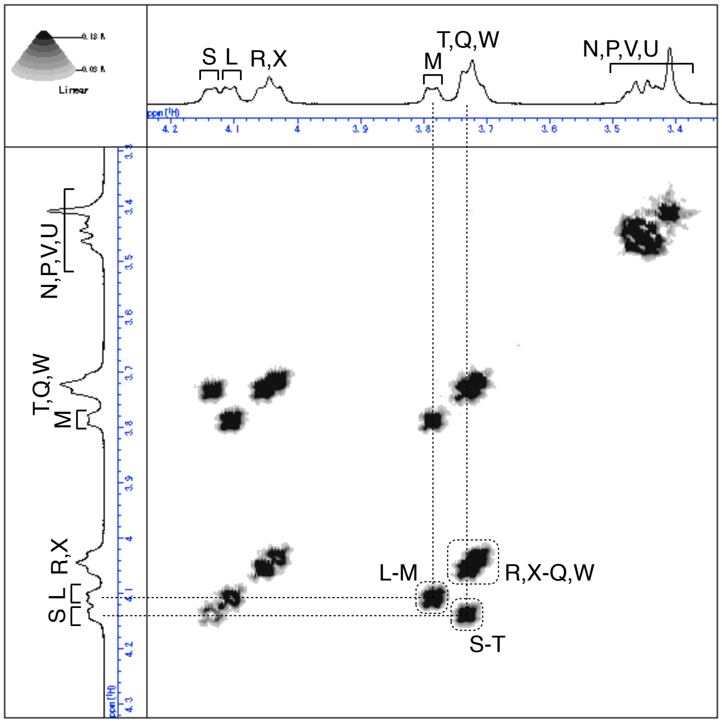

Supplementary Figure S3. Partial HH-COSY correlations of **1-H$_2$** (400 MHz, $CDCl_3$, 298 K).

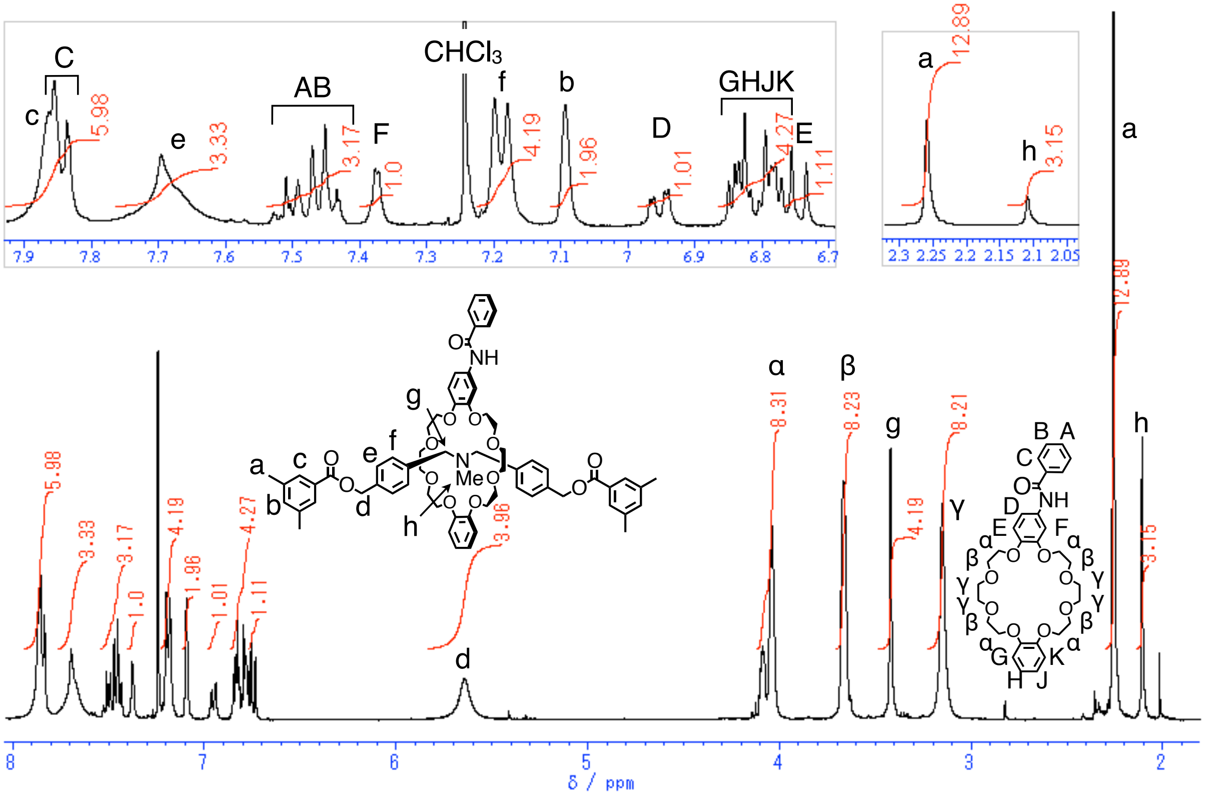

Supplementary Figure S4. ^{1}H NMR spectrum of **1-Me** (400 MHz, $CDCl_3$, 333 K).

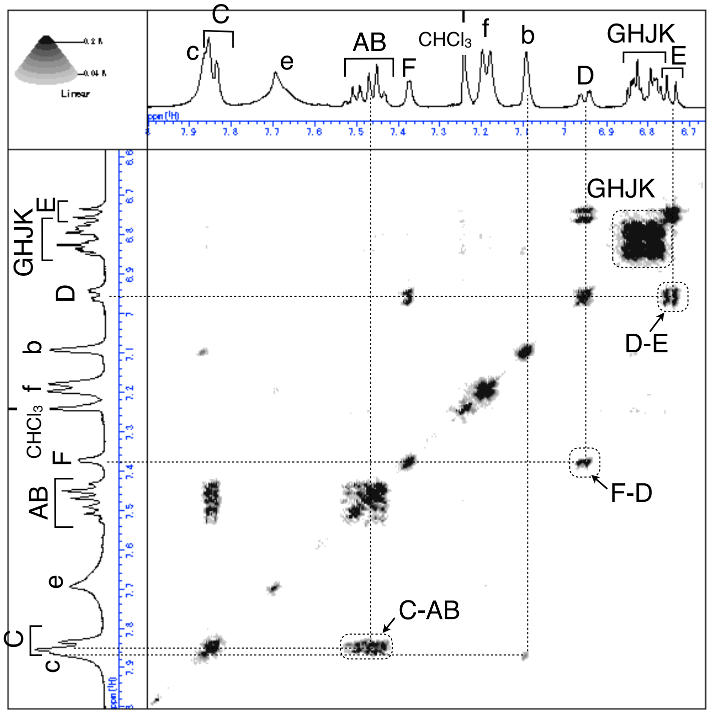

Supplementary Figure S5. Partial HH-COSY correlations of **1-Me** (400 MHz, $CDCl_3$, 333 K).

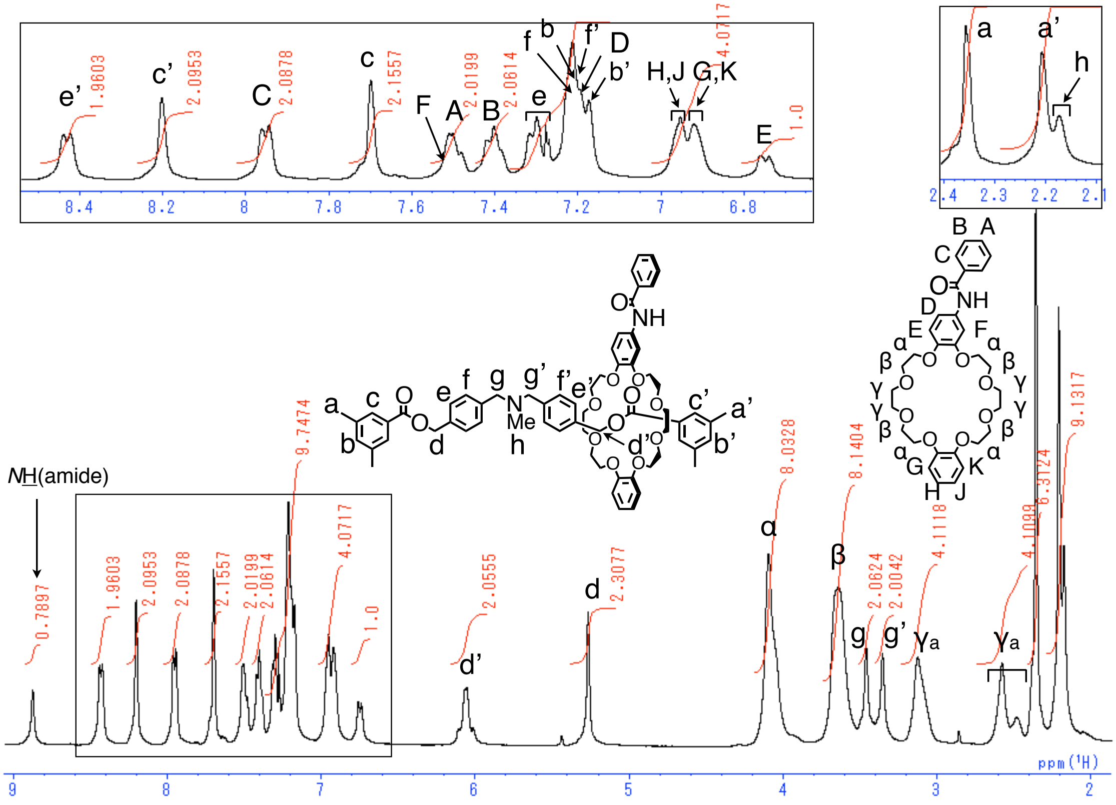

Supplementary Figure S6. ^{1}H NMR spectrum of **1-Me** (400 MHz, $CDCl_3$, 233 K).

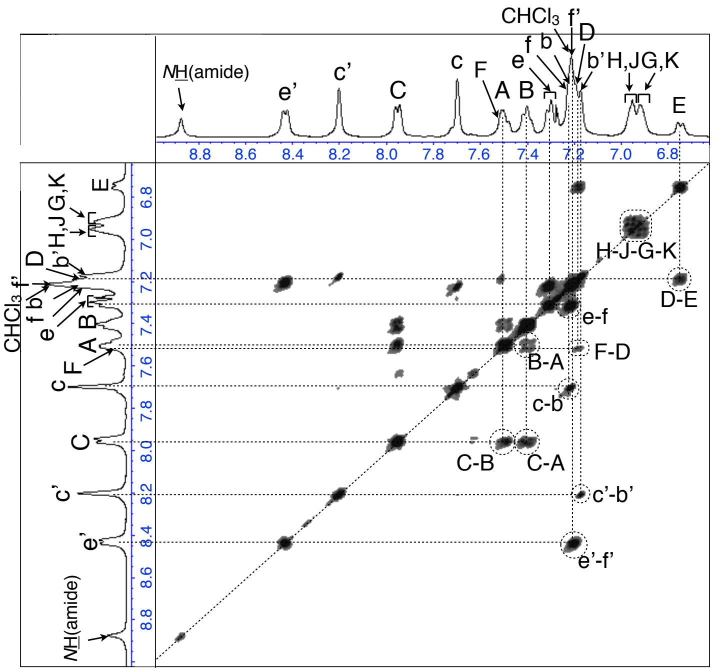

Supplementary Figure S7. Partial HH-COSY correlation of **1-Me** (400 MHz, $CDCl_3$, 233 K).

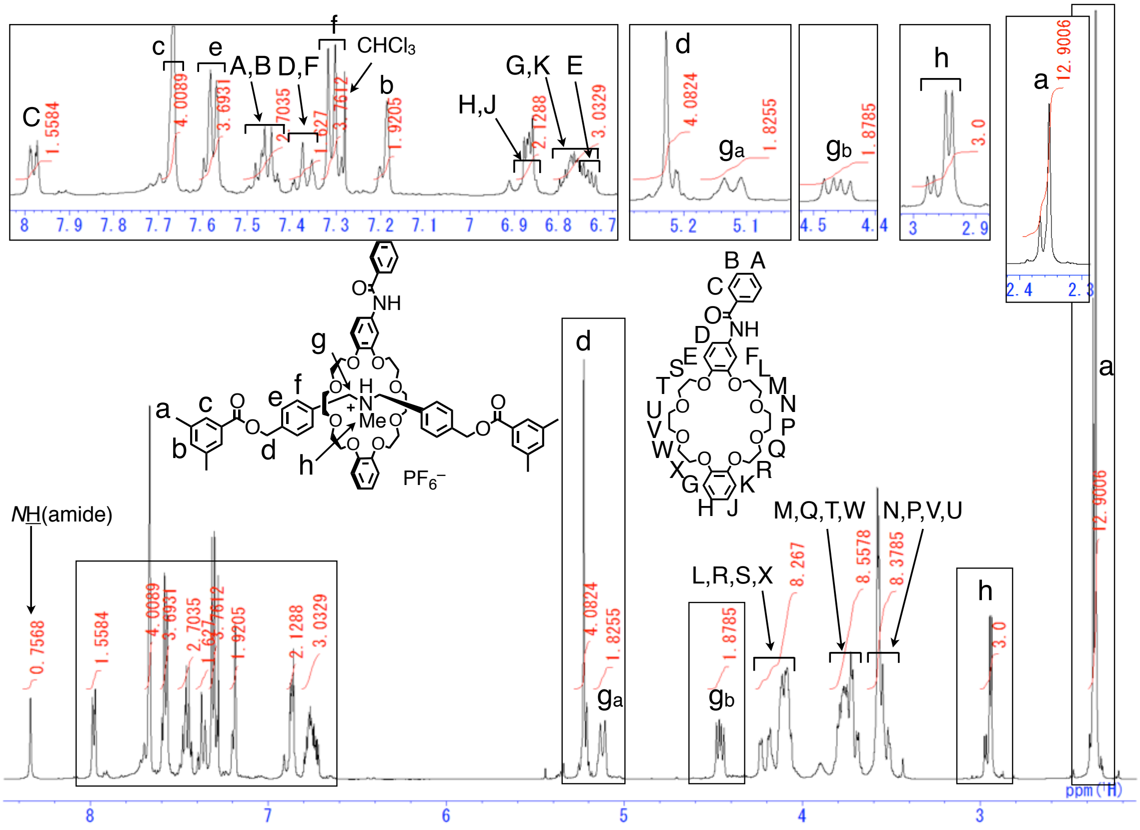

Supplementary Figure S8. ^{1}H NMR spectrum of **1-MeH** (400 MHz, $CDCl_3$, 333 K). The detailed reasons of signal splitting (e.g., protons *h*, *d*, *a*) are still unclear at this stage.

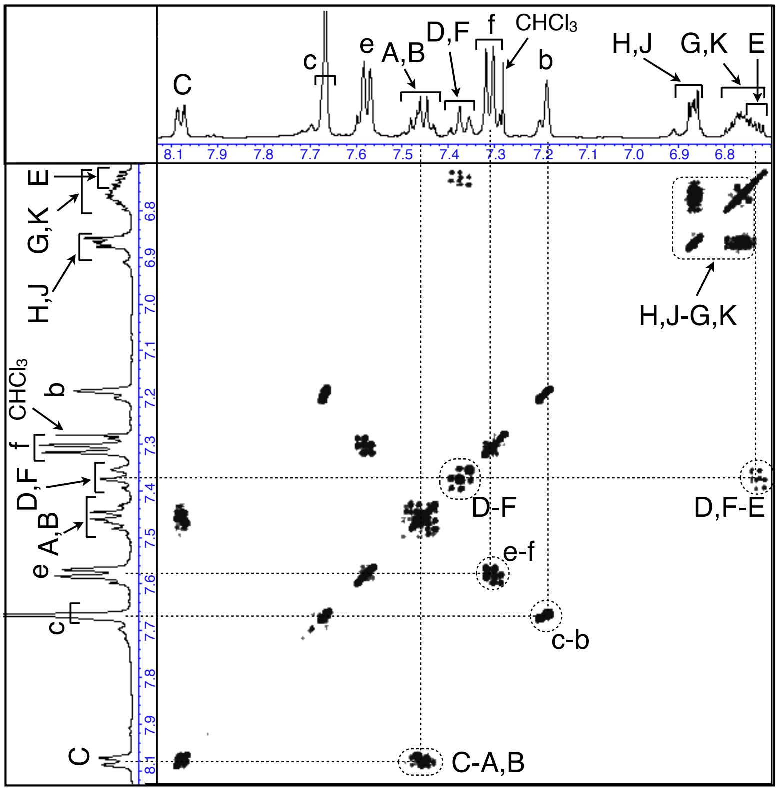

Supplementary Figure S9. Partial HH-COSY correlation of **1-MeH** (400 MHz, $CDCl_3$, 333 K).

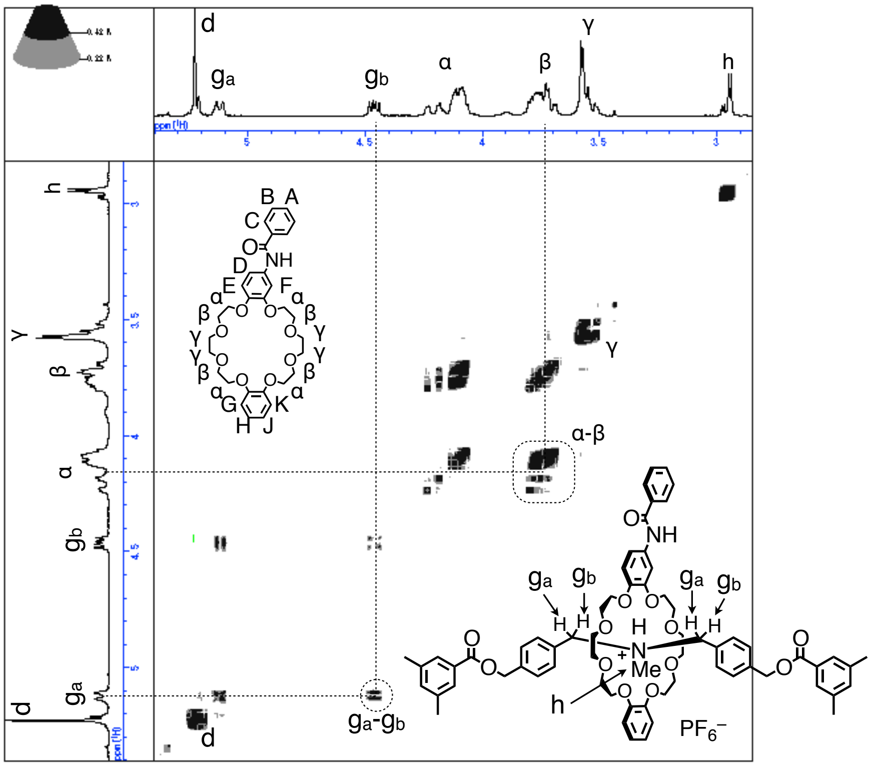

Supplementary Figure S10. Partial HH-COSY correlation of **1-MeH** (400 MHz, $CDCl_3$, 333 K).

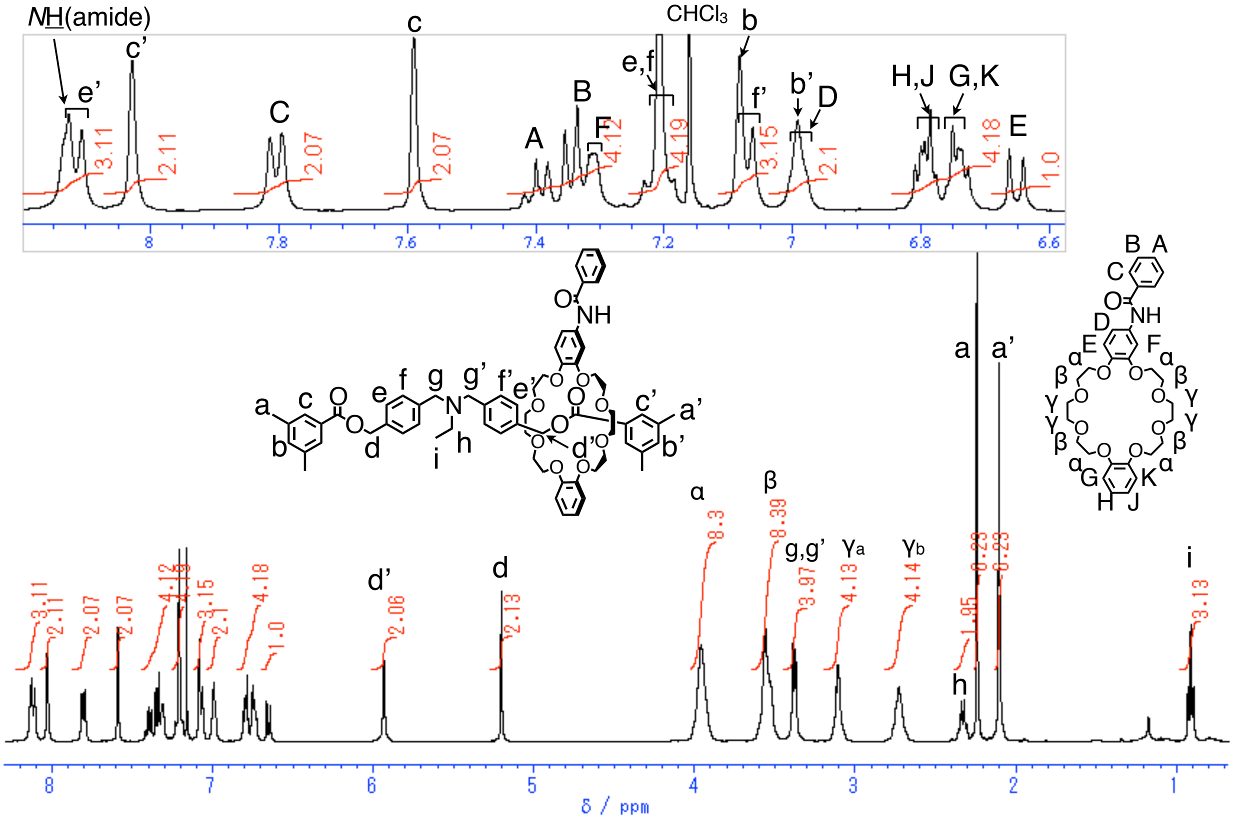

Supplementary Figure S11. ^{1}H NMR spectrum of **1-Et** (400 MHz, $CDCl_3$, 333 K). To prevent splitting of ^{1}H signals due to relatively slow conformational and co-conformational behavior at low temperature, the NMR spectra of **1-Et** were measured at high temperature.

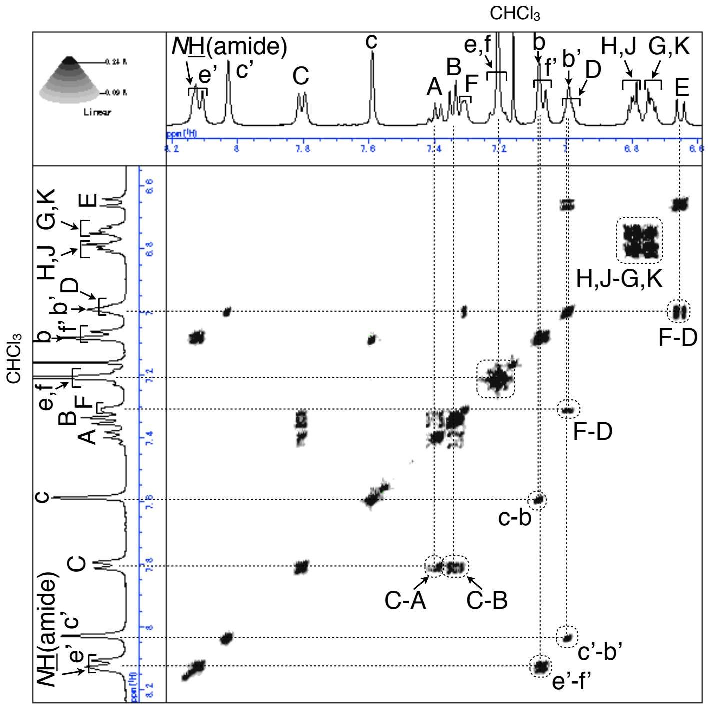

Supplementary Figure S12. Partial HH-COSY correlation of **1-Et** (400 MHz, $CDCl_3$, 333 K).

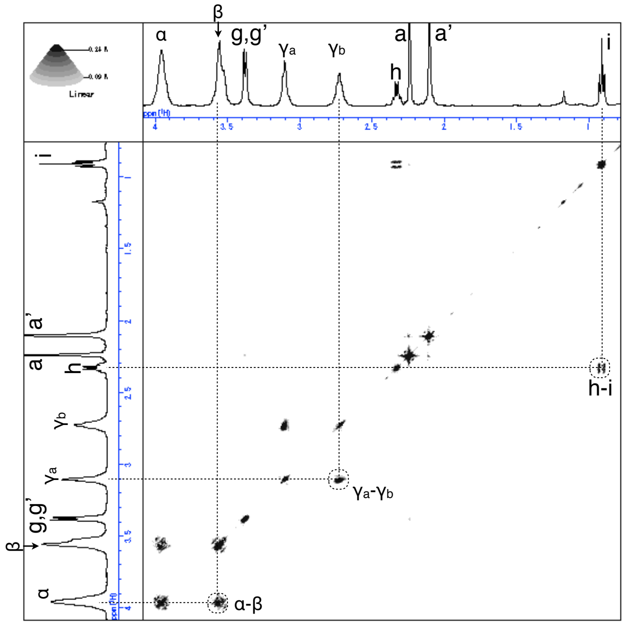

Supplementary Figure S13. Partial HH-COSY correlation of **1-Et** (400 MHz, $CDCl_3$, 333 K).

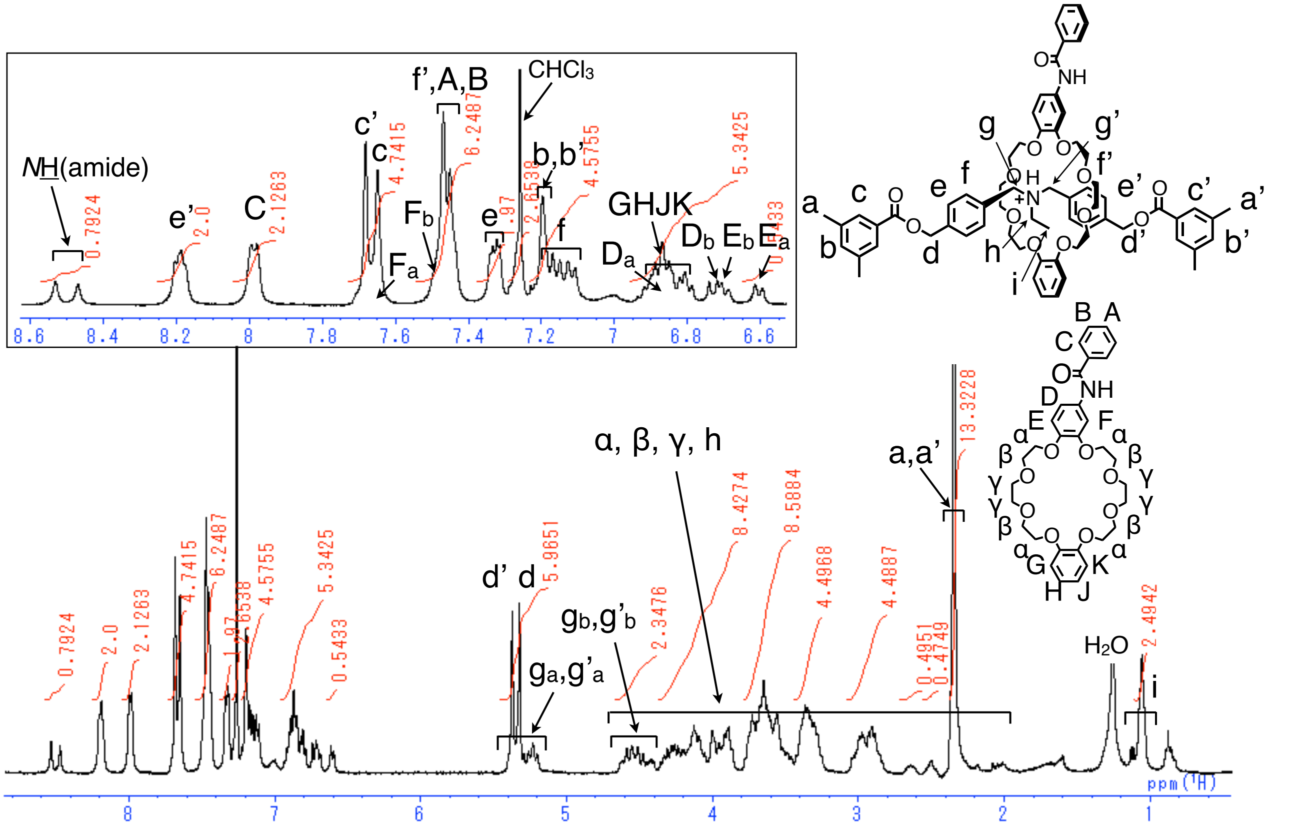

Supplementary Figure S14. ^{1}H NMR spectrum of **1-EtH** (400 MHz, $CDCl_3$, 298 K). The spectrum is complicated mainly due to the diastereomerism from co-conformationally mechanical planar chirality and co-conformationally mechanical point chirality on *N*-atom. However, the complexity of the ^{1}H NMR spectrum of **1-EtH** (*e.g.*, amide *N*H proton) cannot be fully explained at this stage.

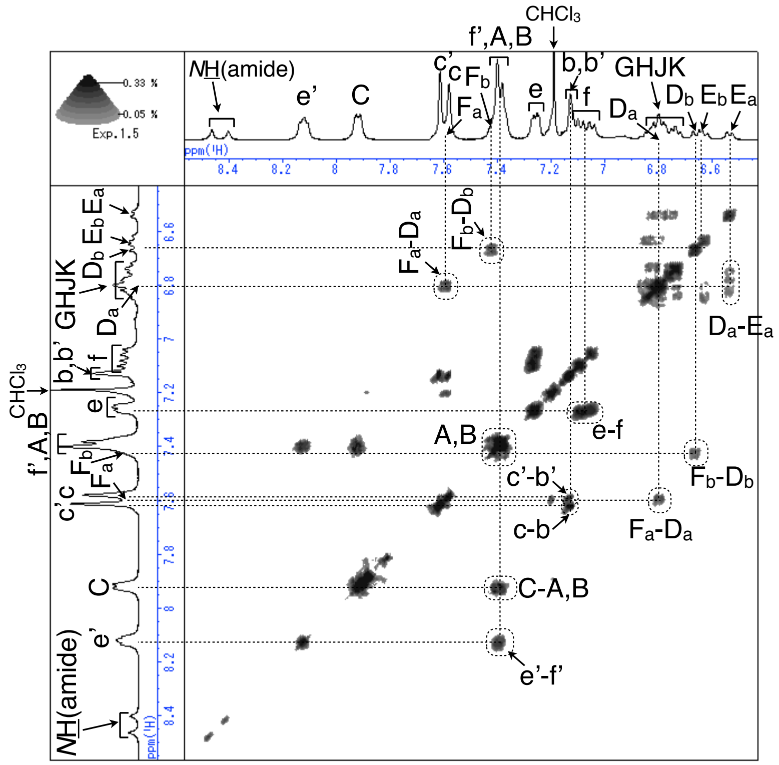

Supplementary Figure S15. Partial HH-COSY correlation of **1-EtH** (400 MHz, $CDCl_3$, 298 K).

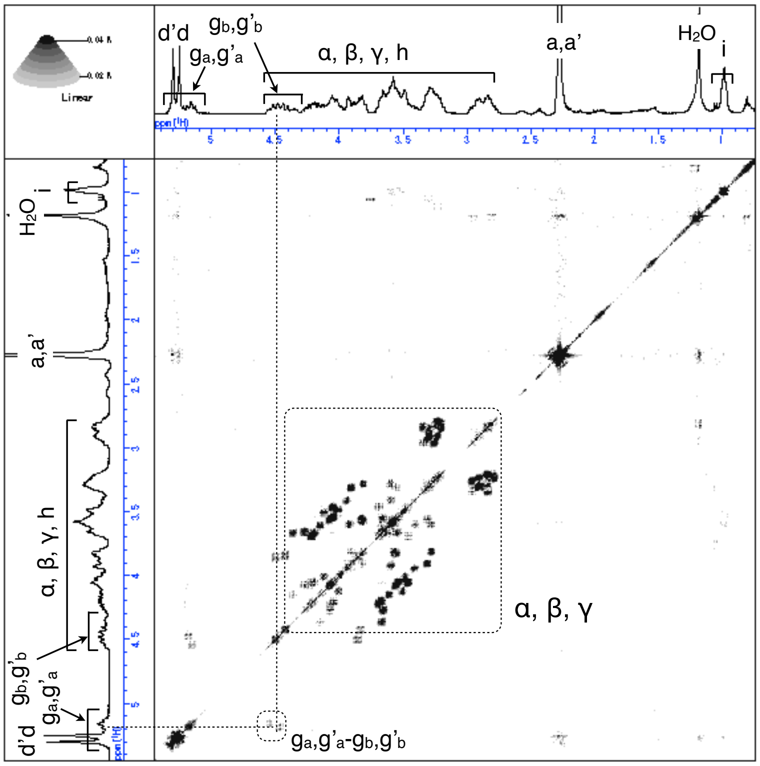

Supplementary Figure S16. Partial HH-COSY correlation of **1-EtH** (400 MHz, $CDCl_3$, 298 K).

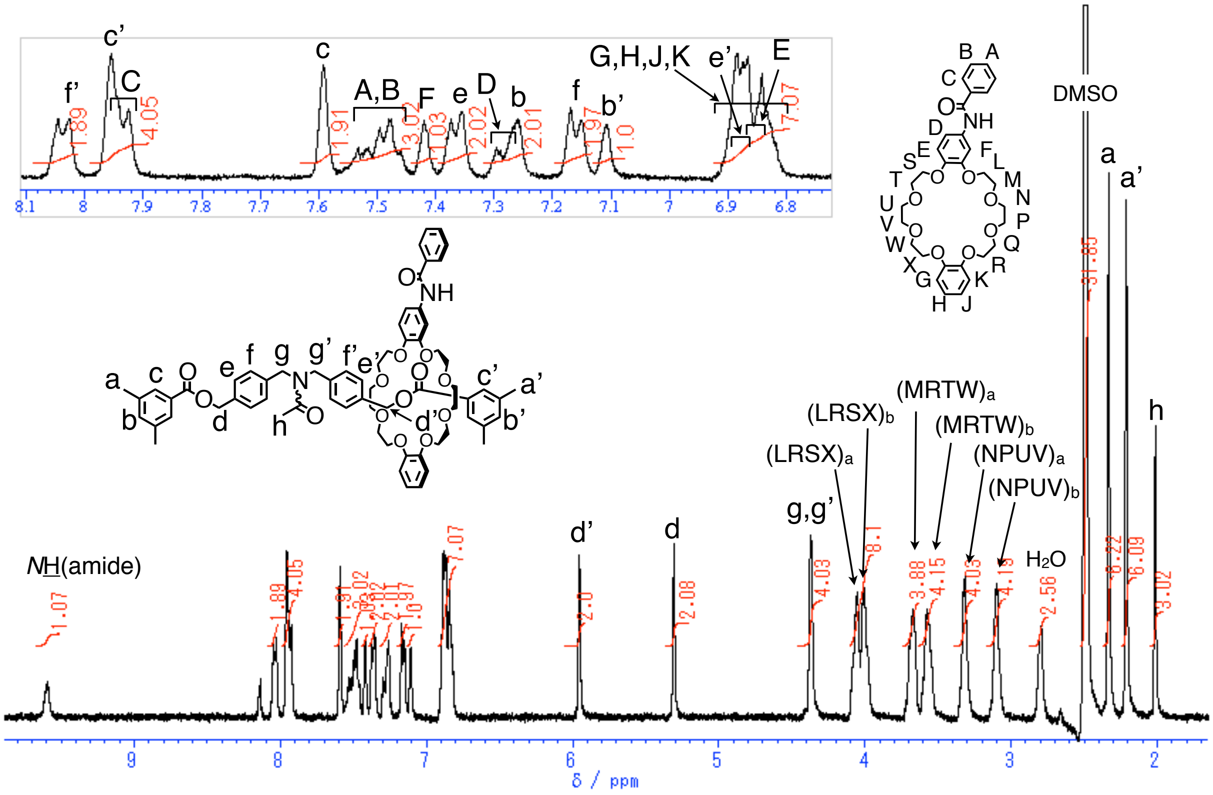

Supplementary Figure S17. ^{1}H NMR spectrum of **1-Ac** (400 MHz, DMSO-d_6, 413 K). To prevent splitting of ^{1}H signals due to slow *S-cis*/*S-trans* isomerization of the tertiary amide group on the axle component at low temperature, the ^{1}H NMR spectrum and HH-COSY correlation (Supplementary Figure S18) of **1-Ac** were measured at high temperature.

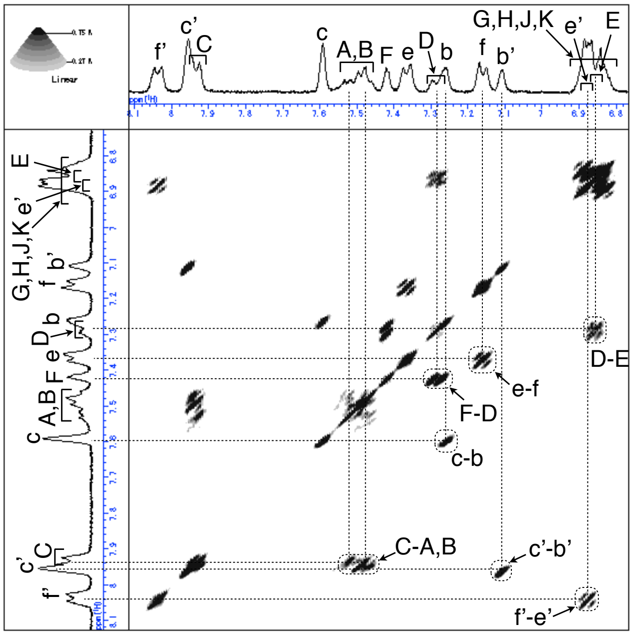

Supplementary Figure S18. Partial HH-COSY correlation of **1-Ac** (400 MHz, DMSO-d_6, 413 K).

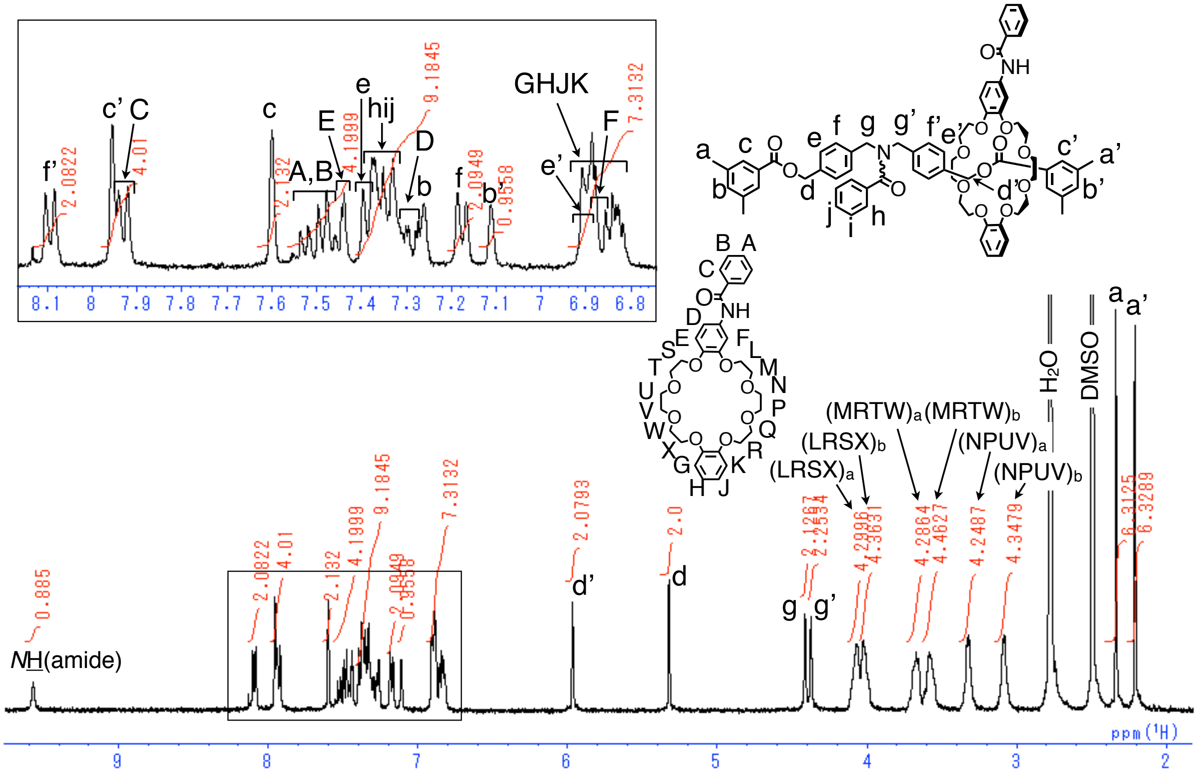

Supplementary Figure S19. ^{1}H NMR spectrum of **1-Bz** (400 MHz, DMSO-d_6, 413 K). To prevent splitting of ^{1}H signals due to slow *S-cis*/*S-trans* isomerization of the tertiary amide group on the axle component at low temperature, the ^{1}H NMR spectrum and HH-COSY correlation (Supplementary Figure S20) of **1-Bz** were measured at high temperature.

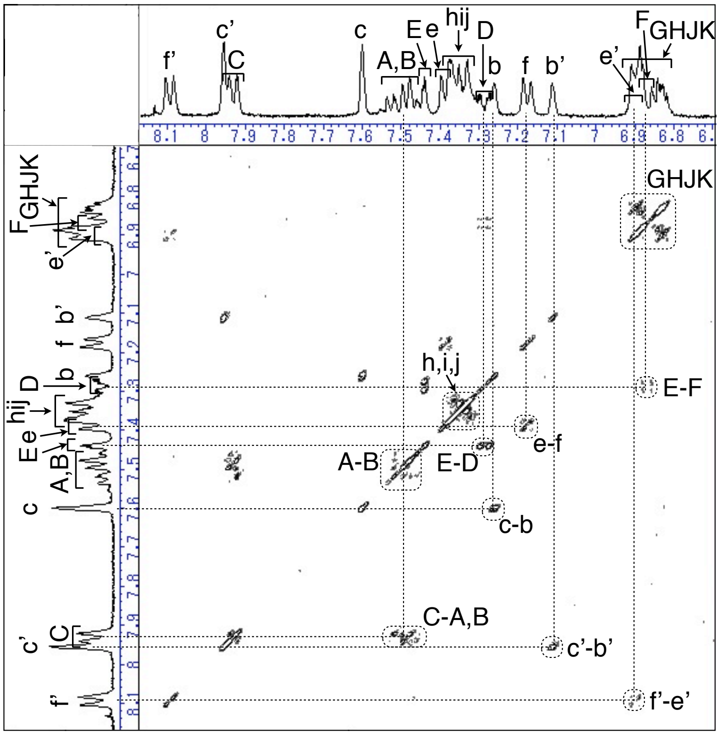

Supplementary Figure S20. Partial HH-COSY correlation of **1-Bz** (400 MHz, DMSO-d_6, 413 K).

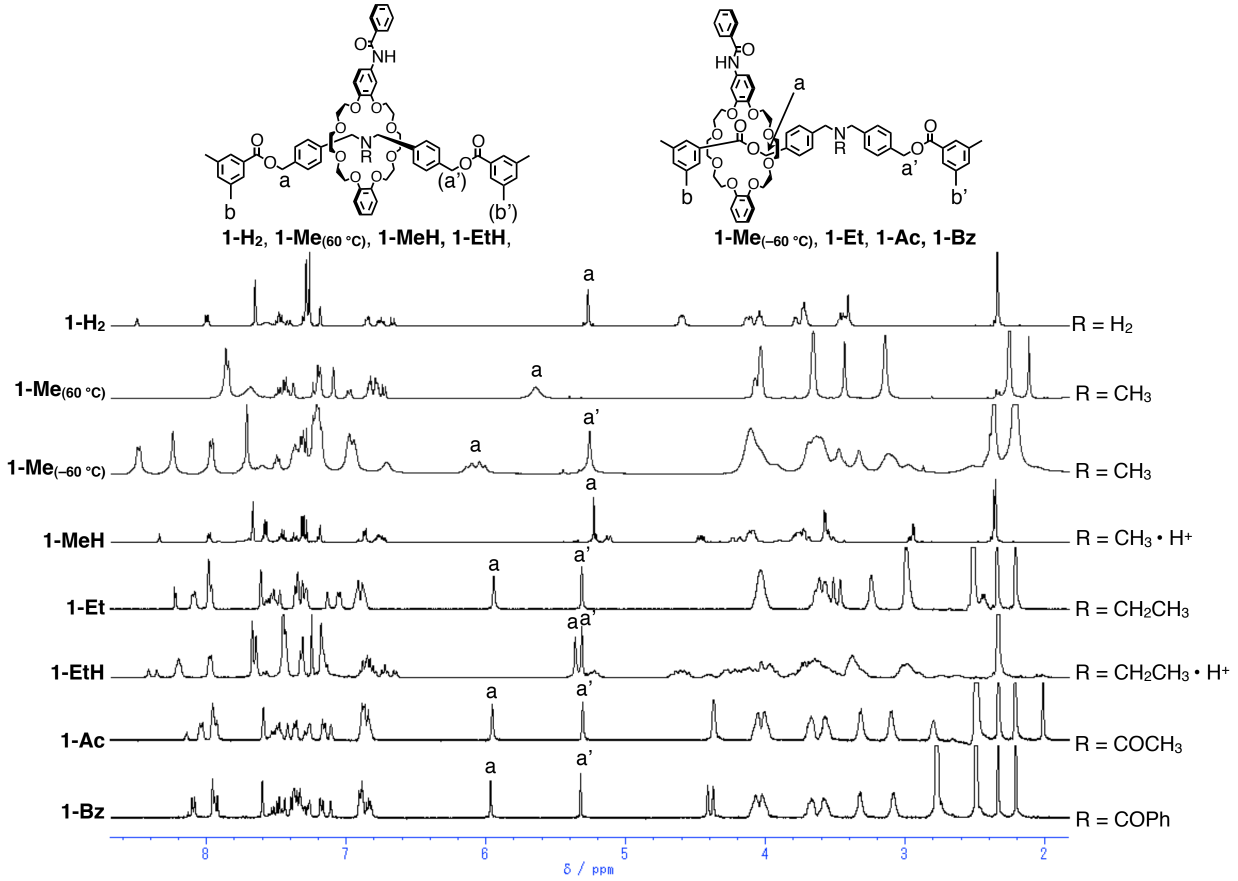

Supplementary Figure S21. ^{1}H NMR (400 MHz) spectra of **1-H$_2$** ($CDCl_3$, 298 K), **1-Me** ($CDCl_3$, 333 and 213 K), **1-MeH** ($CDCl_3$, 333K), **1-Et** (DMSO-d_6, 373 K), **1-EtH** ($CDCl_3$, 333K), **1-Ac** (DMSO-d_6, 413 K) and **1-Bz** (DMSO-d_6, 413 K).

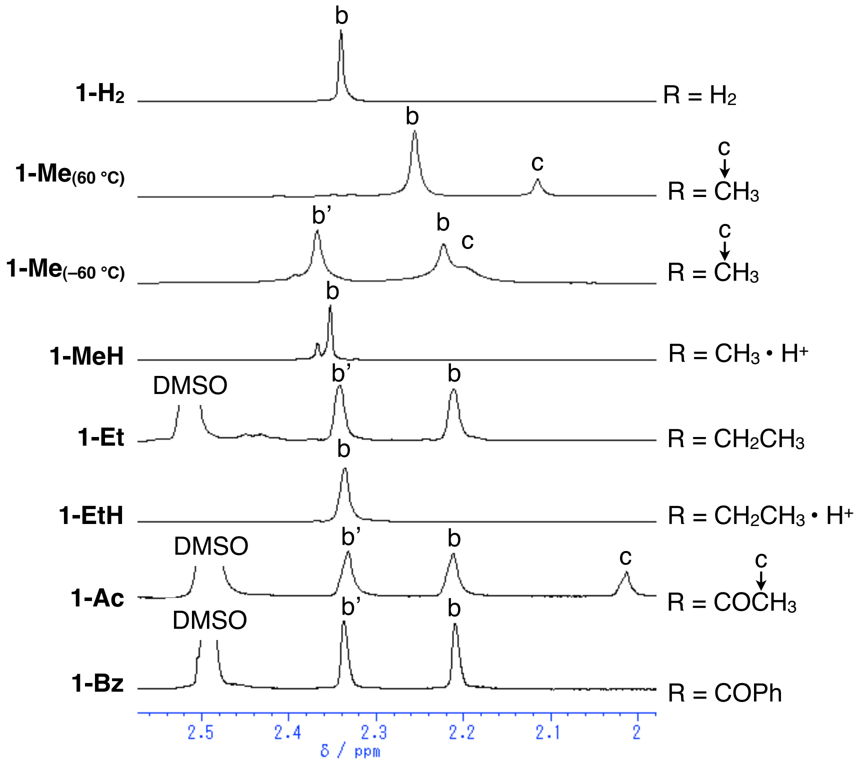

Supplementary Figure S22. Partial ^1H NMR (400 MHz) spectra of **1-H₂** (CDCl₃, 298 K), **1-Me** (CDCl₃, 333 and 213 K), **1-MeH** (CDCl₃, 333K), **1-Et** (DMSO-d_6, 373 K), **1-EtH** (CDCl₃, 333K), **1-Ac** (DMSO-d_6, 413 K) and **1-Bz** (DMSO-d_6, 413 K).

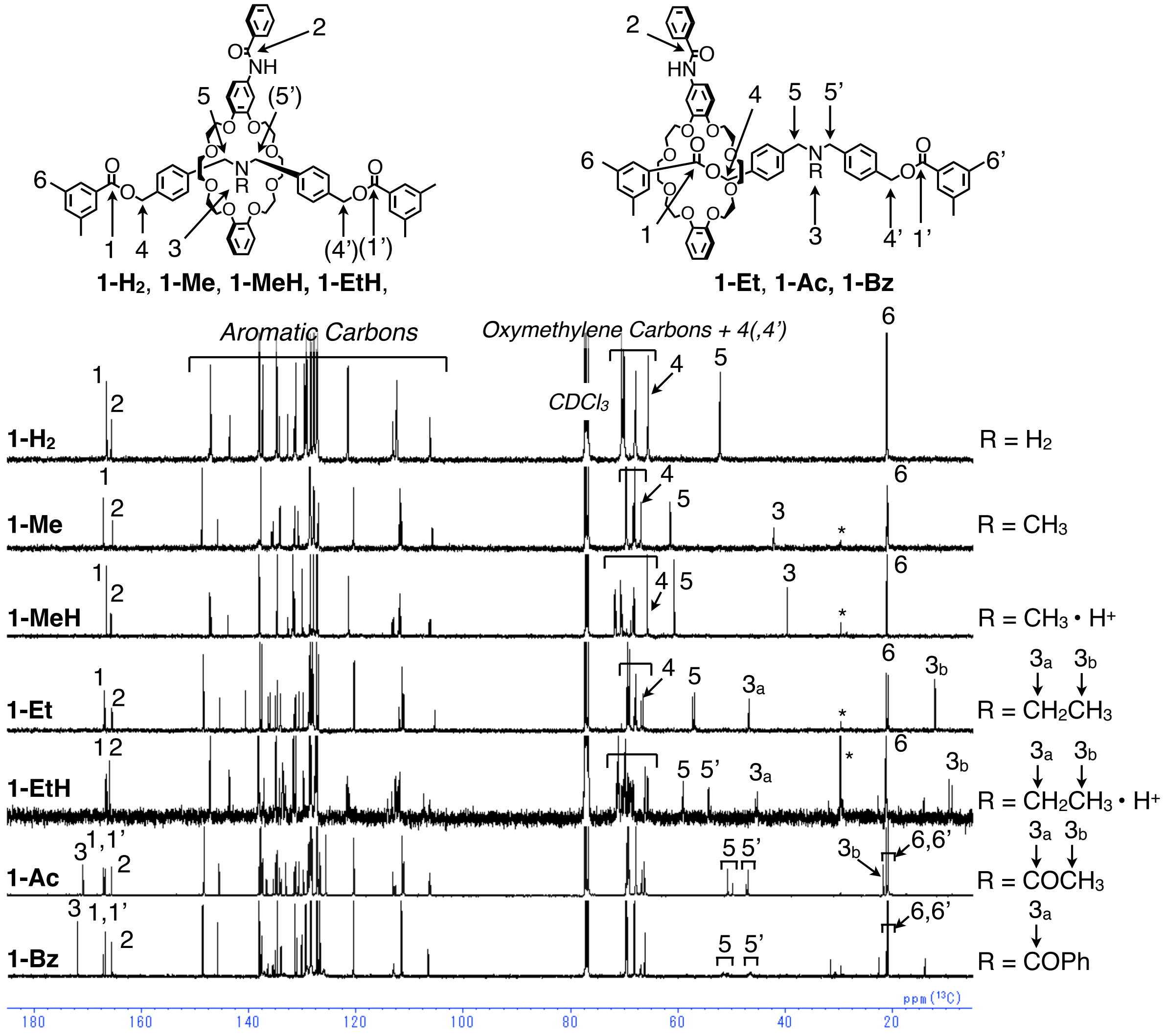

Supplementary Figure S23. ^{13}C NMR spectra of **1-H$_2$**, **1-Me**, **1-MeH**, **1-Et**, **1-EtH**, **1-Ac** and **1-Bz** (400 MHz, $CDCl_3$, 333 K).

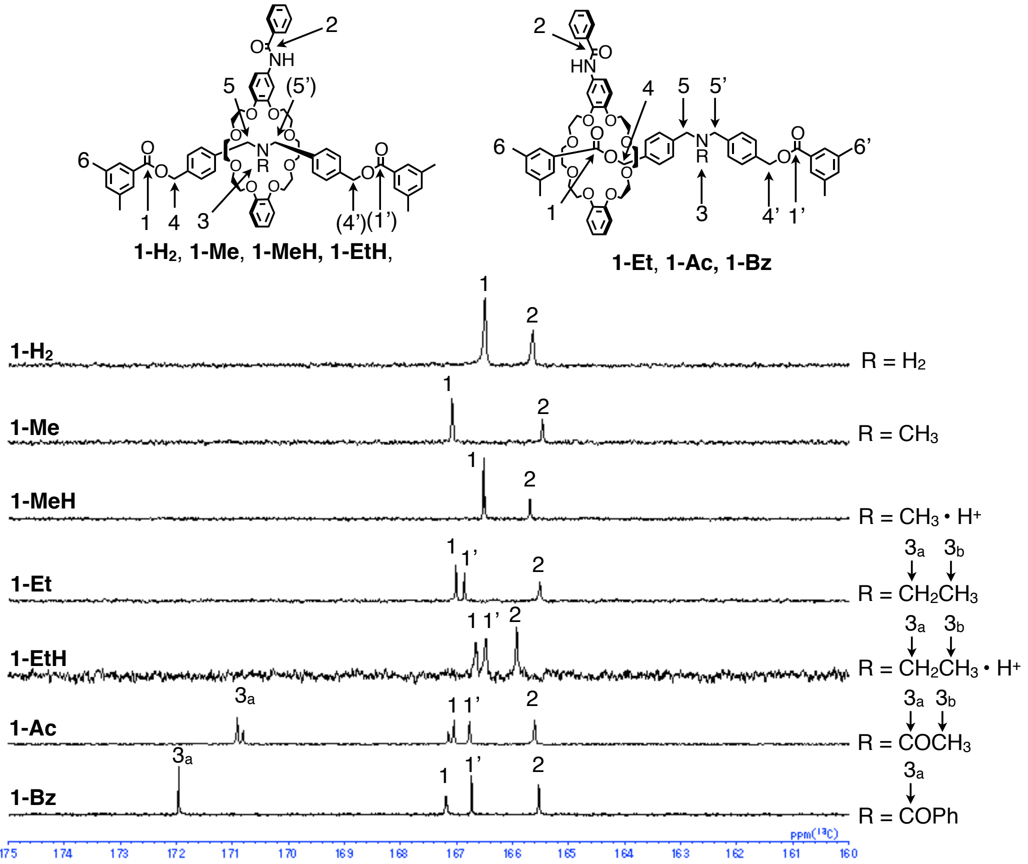

Supplementary Figure S24. Partial ^{13}C NMR spectra of **1-H*2***, **1-Me**, **1-MeH**, **1-Et**, **1-EtH**, **1-Ac** and **1-Bz** (400 MHz, $CDCl_3$, 333 K).

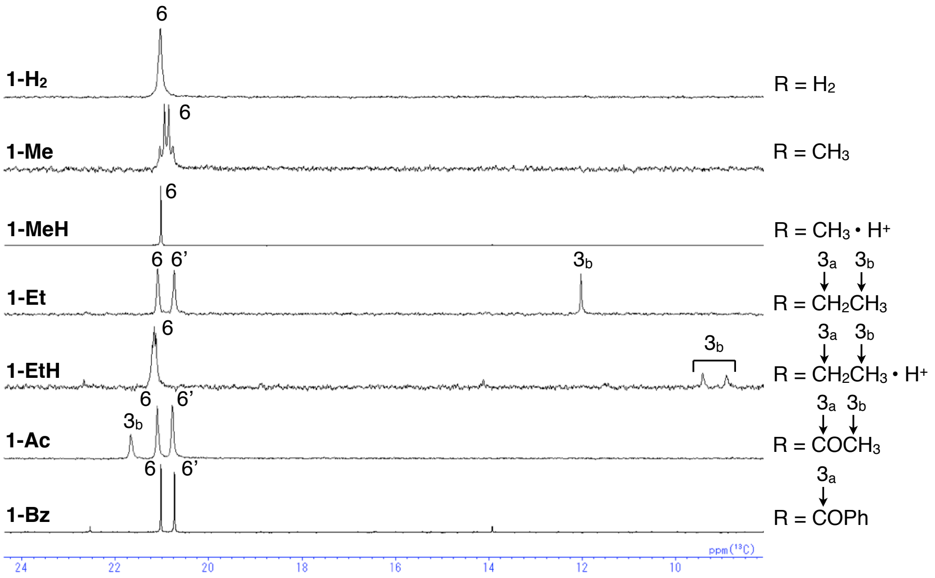

Supplementary Figure S25. Partial ^{13}C NMR spectra of **1-H*2***, **1-Me**, **1-MeH**, **1-Et**, **1-EtH**, **1-Ac** and **1-Bz** (400 MHz, $CDCl_3$, 333 K).

2. IR Spectra

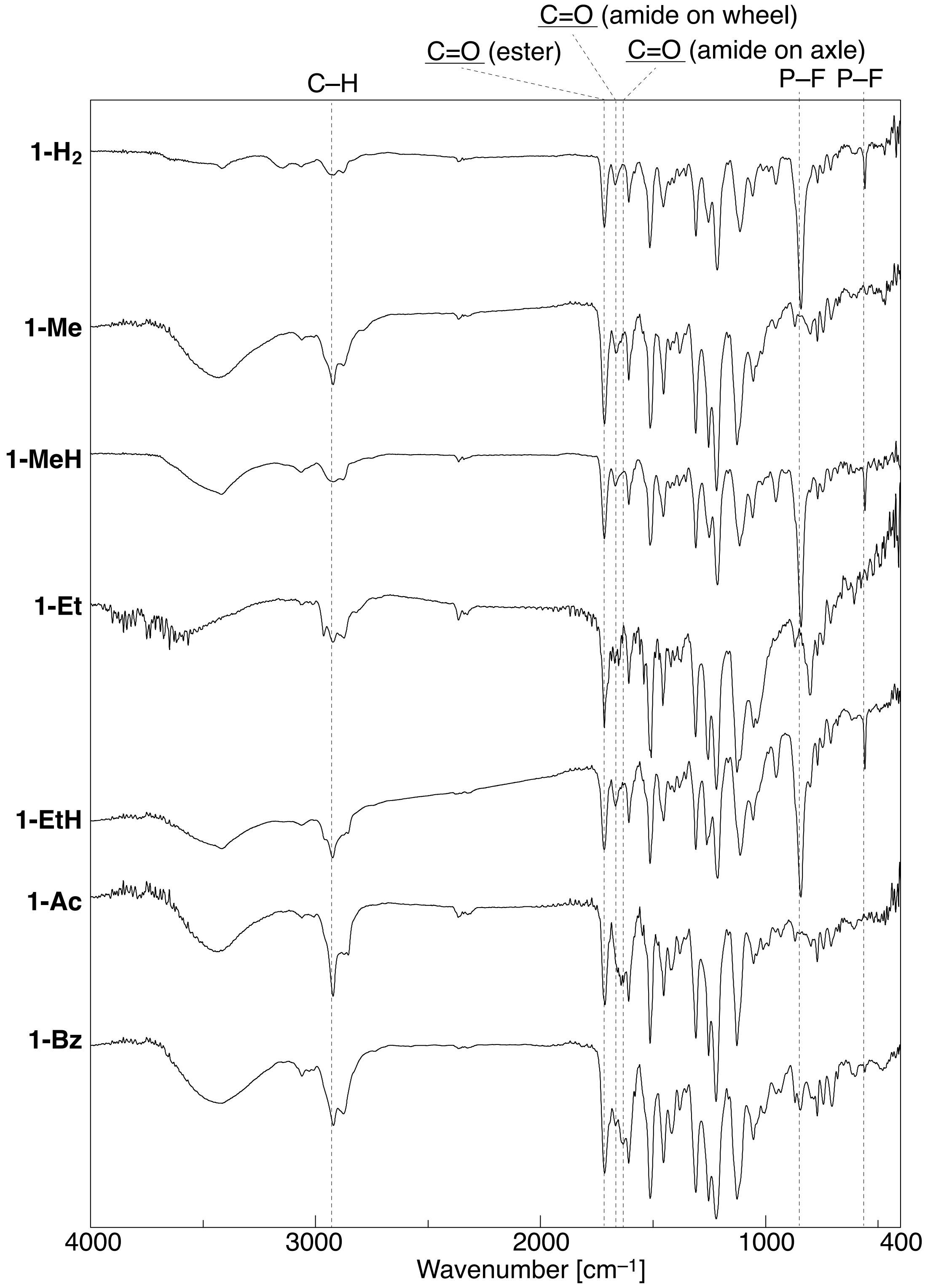

Supplementary Figure S26. FT-IR spectra of **1-*H₂***, **1-Me**, **1-MeH**, **1-Et**, **1-EtH**, **1-Ac** and **1-Bz** (KBr).

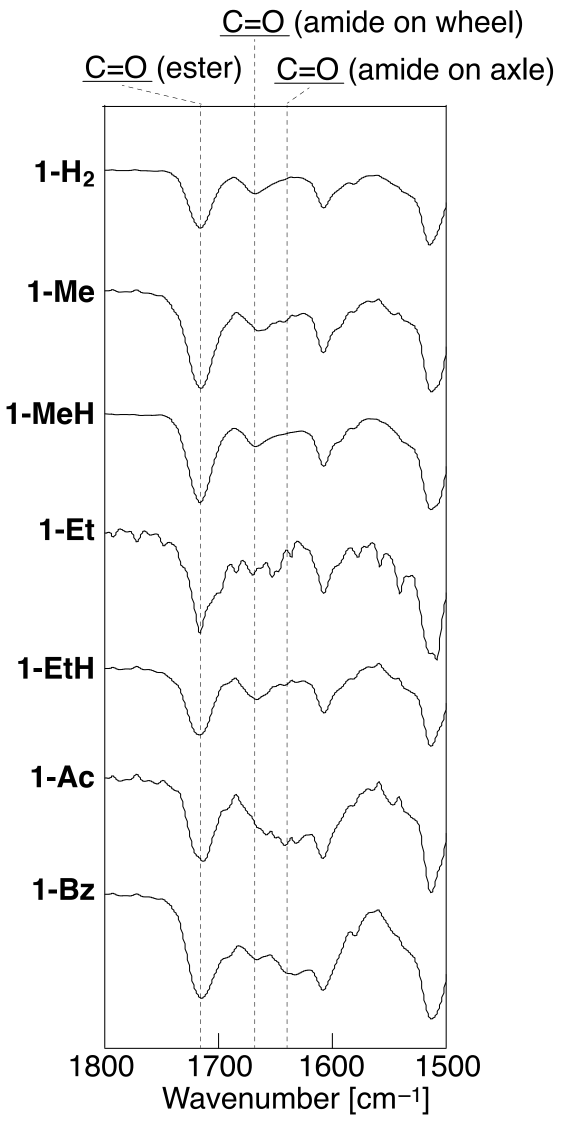

Supplementary Figure S27. Partial FT-IR spectra of **1-H**$_2$, **1-Me**, **1-MeH**, **1-Et**, **1-EtH**, **1-Ac** and **1-Bz** (KBr).

3. High-Resolution Mass Spectra

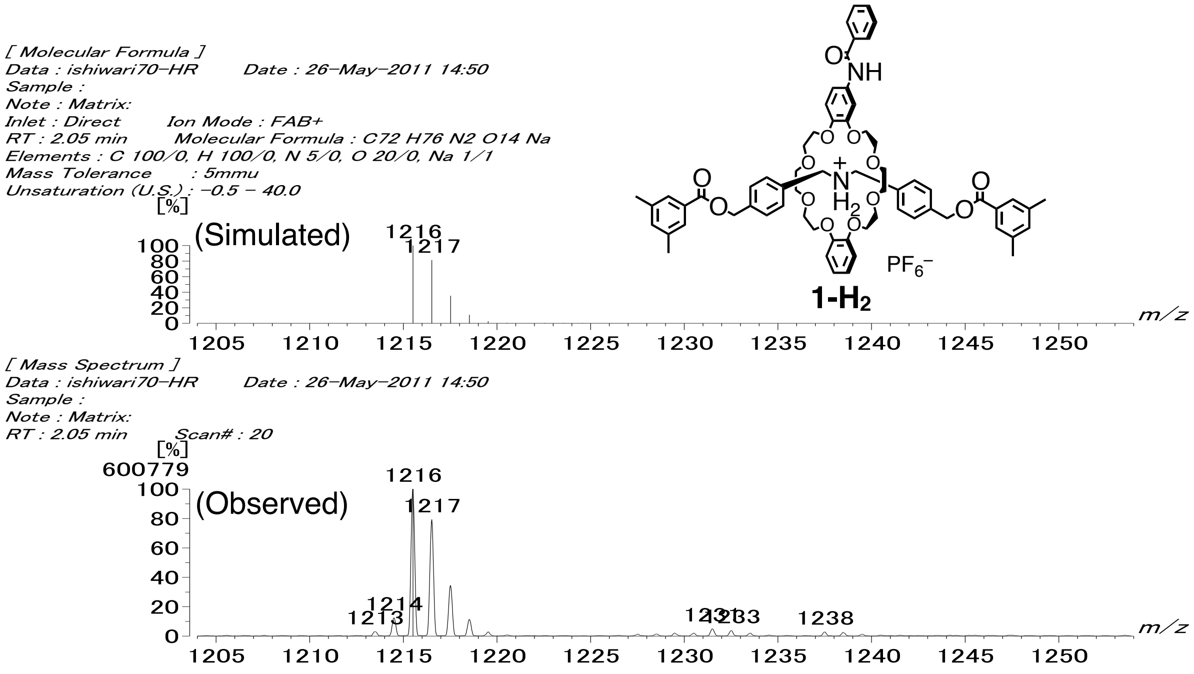

Supplementary Figure S28. HRMS spectrum of **1-H$_2$** (ESI).

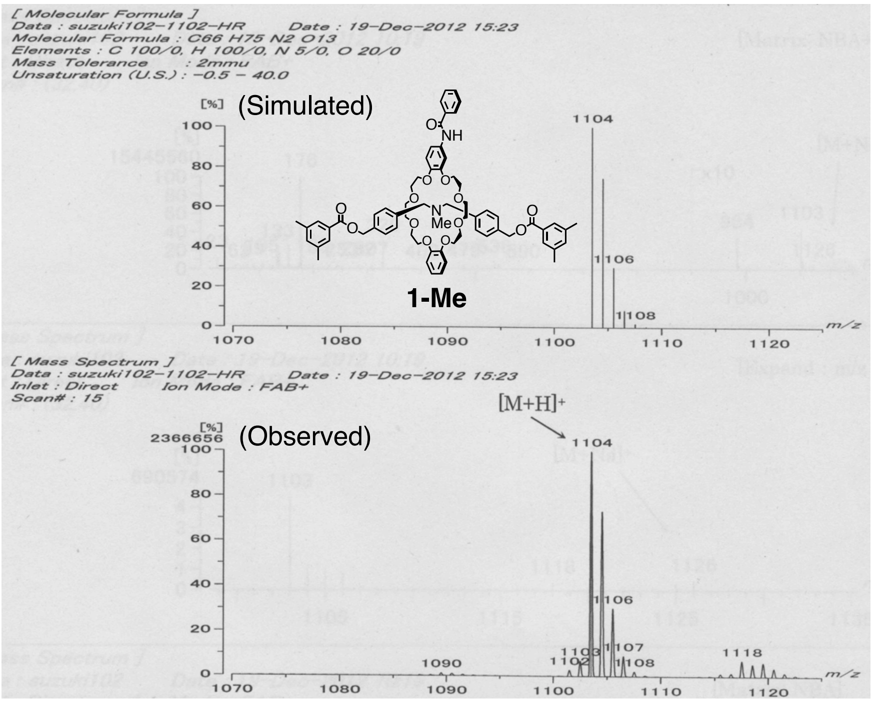

Supplementary Figure S29. HRMS spectrum of **1-Me** (FAB).

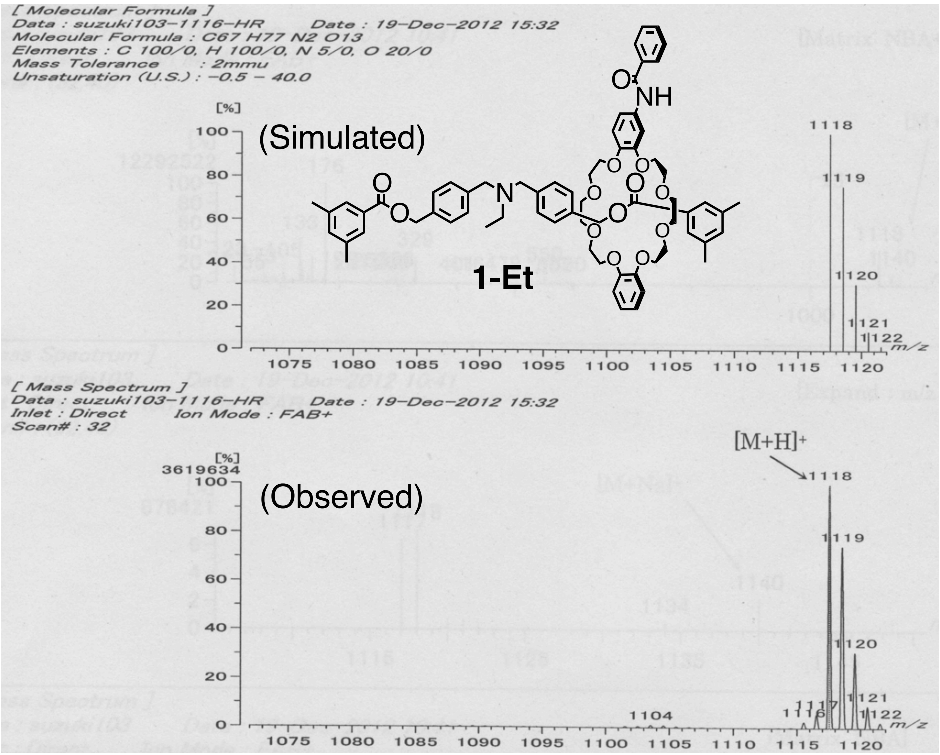

Supplementary Figure S30. HRMS spectrum of **1-Et** (FAB).

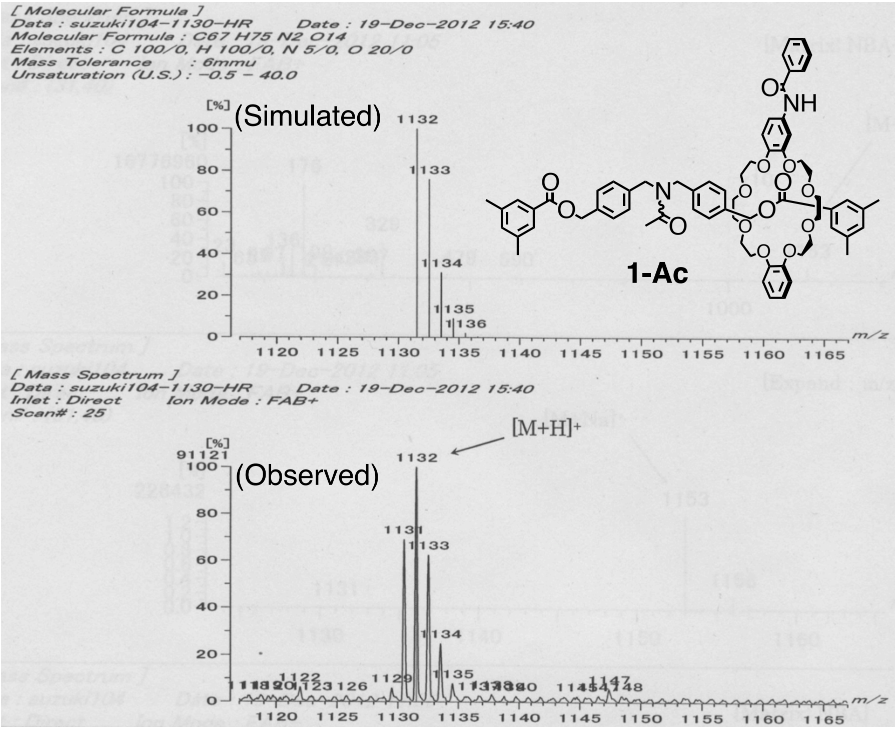

Supplementary Figure S31. HRMS spectrum of **1-Ac** (FAB).

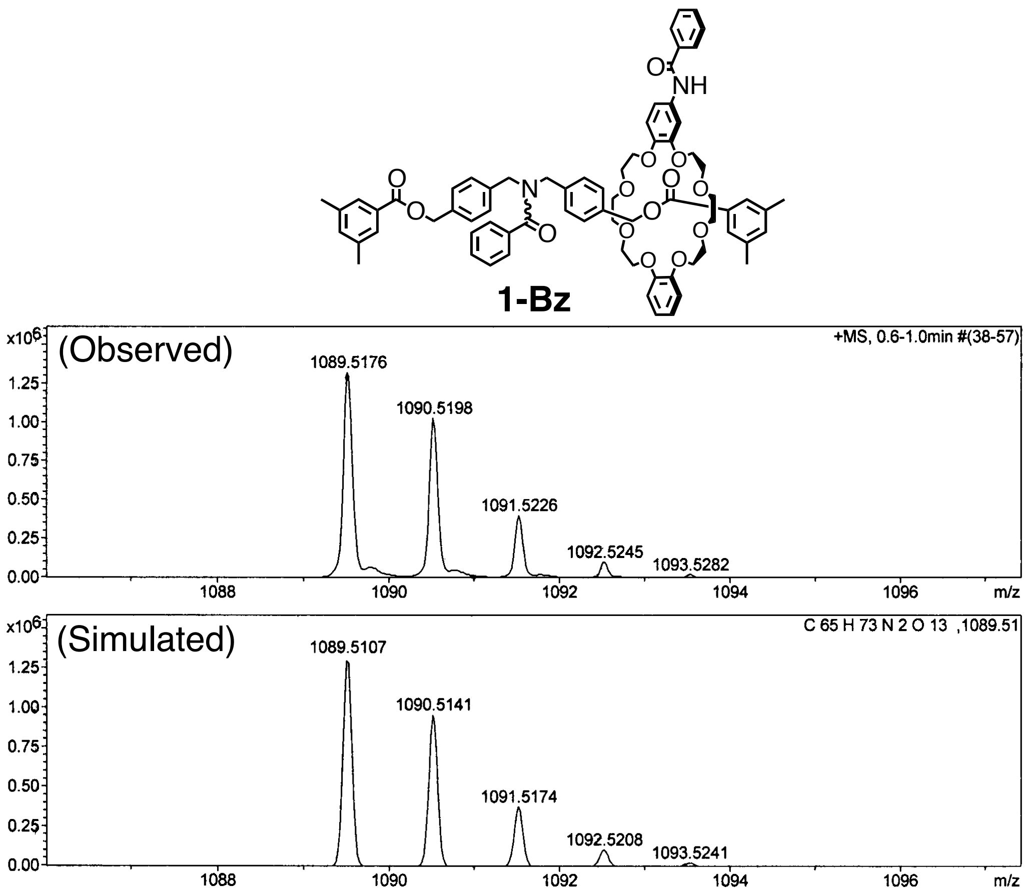

Supplementary Figure S32. HRMS spectrum of **1-Bz** (ESI).

4. Chiral HPLC Profiles

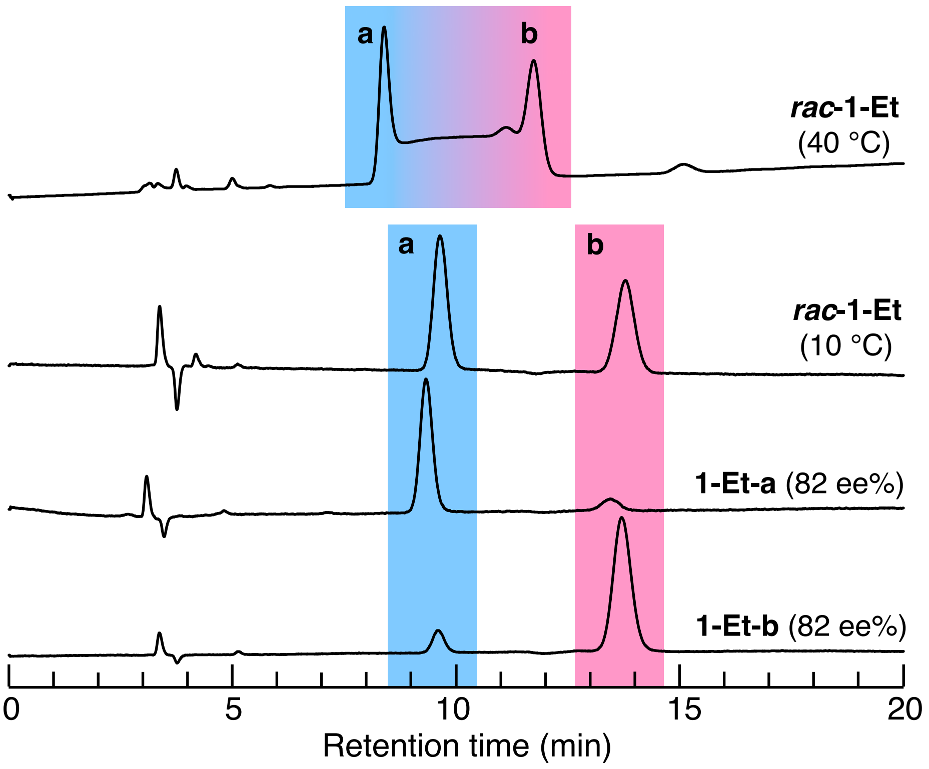

Supplementary Figure S33. Chiral HPLC profiles (CHIRALPAK IA, eluents: *n*-hexane/$CHCl_3$/Et_2NH = 1/1/0.005, isocratic, flow rate = 1.0 mL/min) of ***rac*-1-Et** (at 313 K and 283 K), **1-Et-a** (at 283 K) and **1-Et-b** (at 283 K). Enantiomeric excess of **1-Et-a** and **1-Et-b** (ee%) are calculated from peak areas to be 82% and 82% (), respectively.

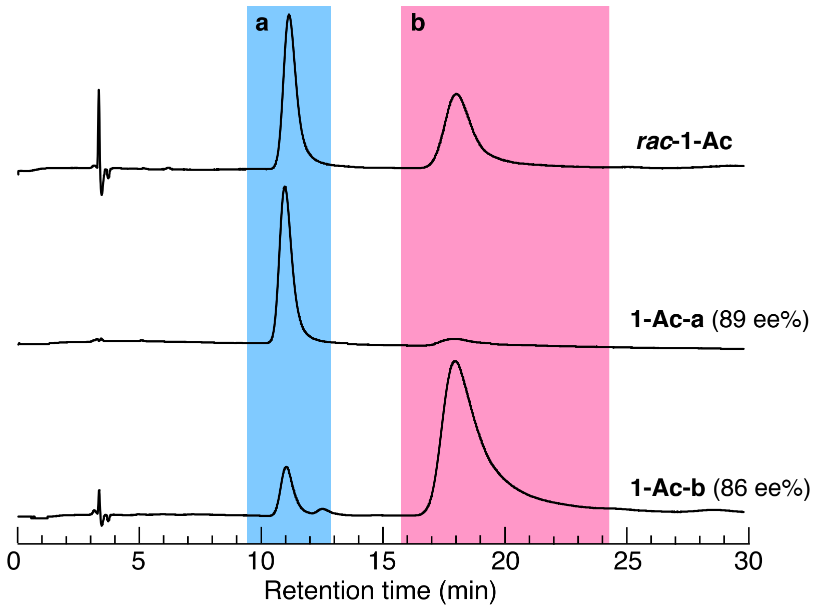

Supplementary Figure S34. Chiral HPLC profiles of ***rac*-1-Ac**, **1-Ac-a** and **1-Ac-b** (CHIRALPAK IA, eluents: *n*-hexane/$CHCl_3$ = 1/1, isocratic, flow rate = 1.0 mL/min, 283 K). Enantiomeric excess of **1-Ac-a** and **1-Ac-b** (ee%) are calculated from peak areas to be 89% and 86%, respectively.

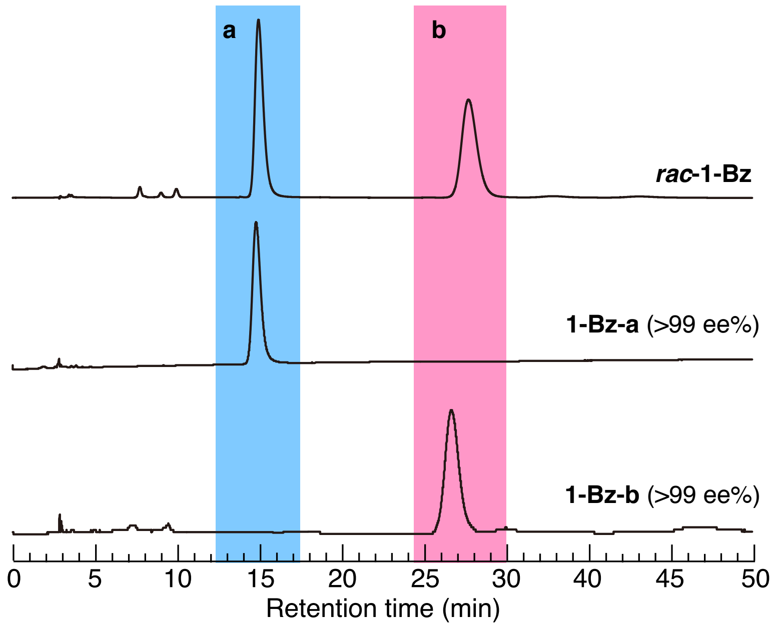

Supplementary Figure S35. Chiral HPLC profiles of ***rac*-1-Bz**, **1-Bz-a** and **1-Bz-b** (CHIRALPAK IA, eluents: *n*-hexane/$CHCl_3$ = 1/1, isocratic, flow rate = 1.0 mL/min, 283 K). Enantiomeric excess of **1-Bz-a** and **1-Bz-b** (ee%) are calculated to be >99% and >99%, respectively.

5. Analysis of Racemization Behaviors

Thermodynamic parameters of racemization of 1-Me were obtained by coalescence method using VT-NMR spectra (Supplementary Figures S36–39).

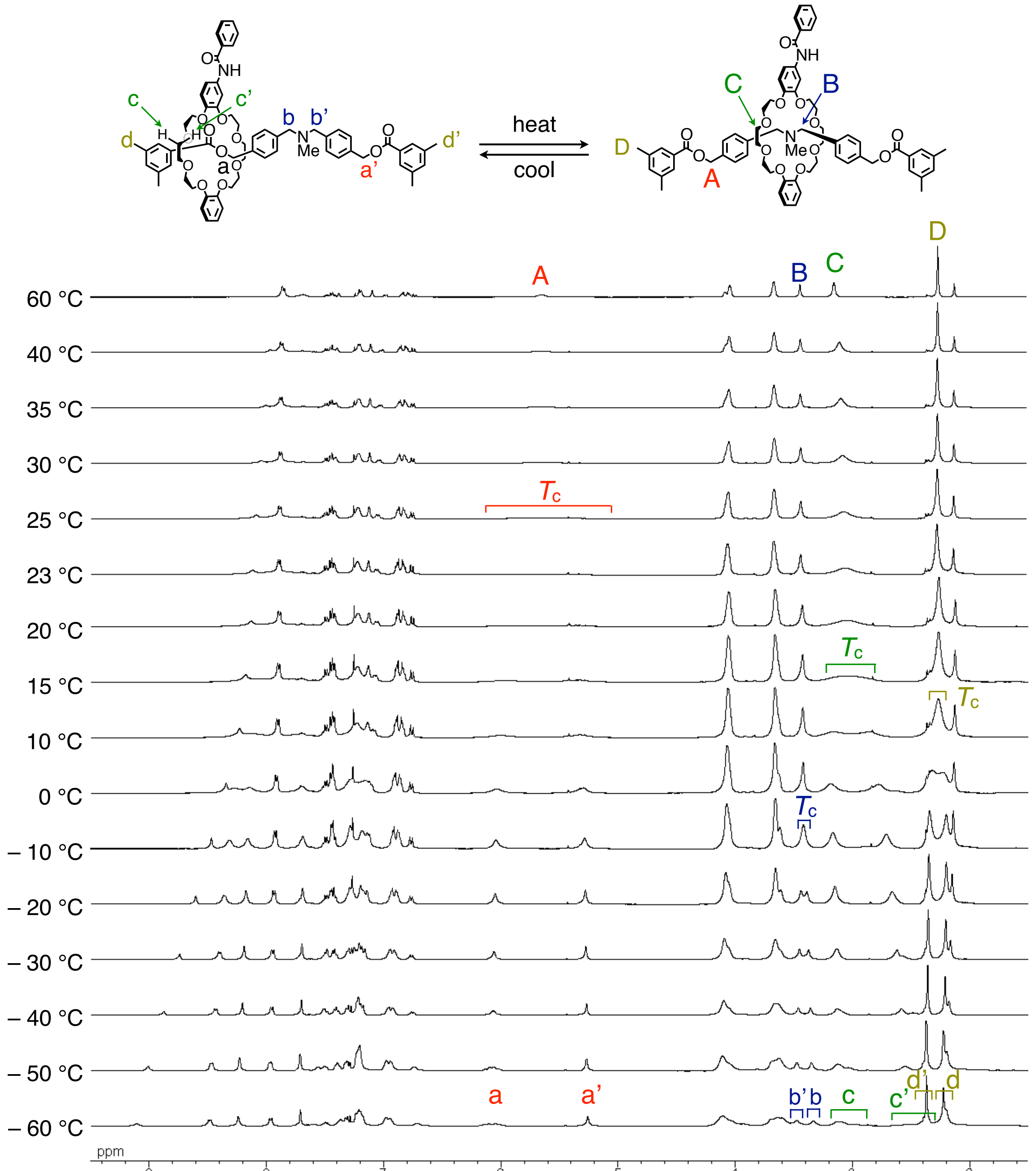

Supplementary Figure S36. VT-^{1}H NMR spectra of **1-Me** (400 MHz, $CDCl_3$, 213–333 K).

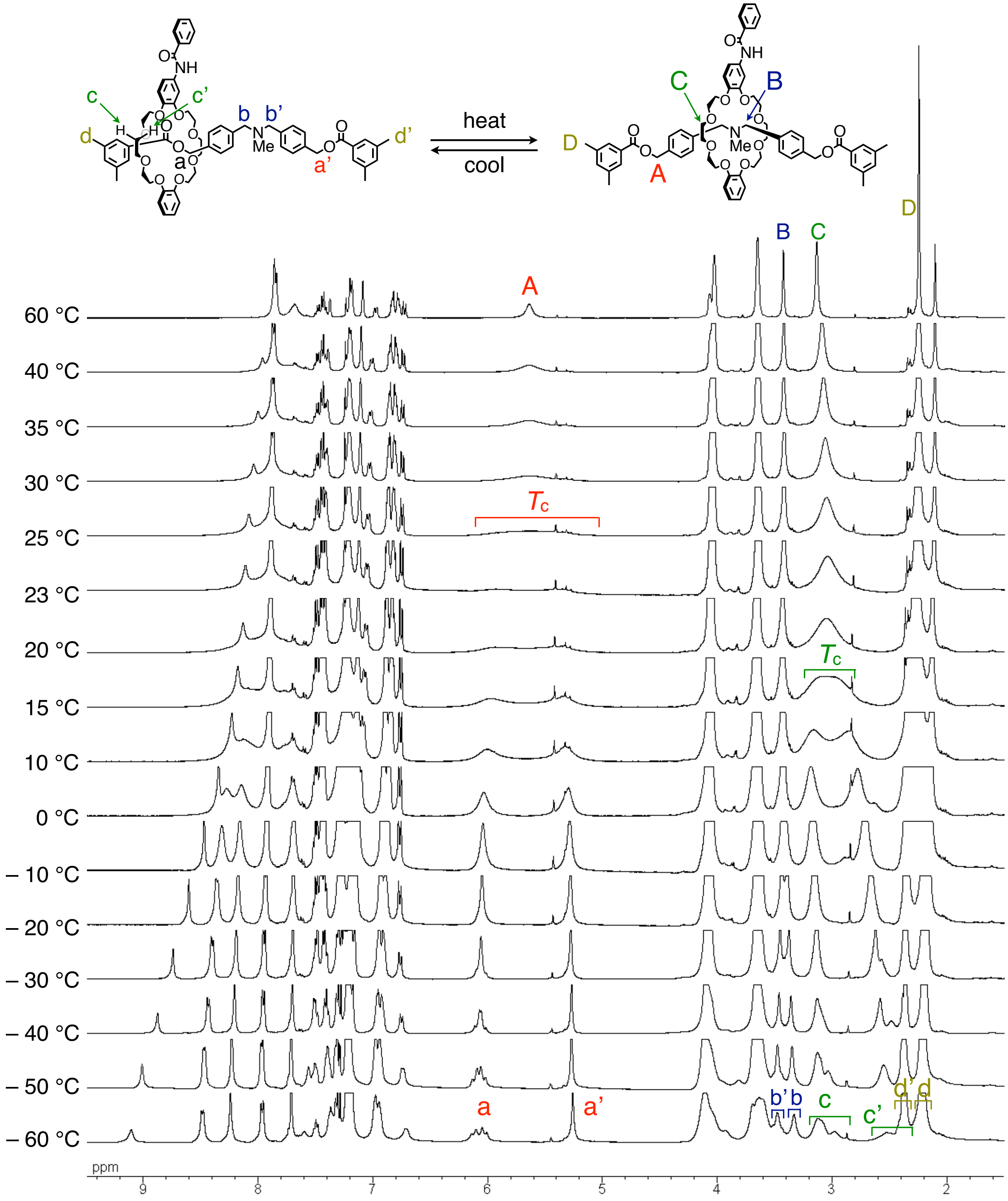

Supplementary Figure S37. Expanded VT-^{1}H NMR spectra of **1-Me** (400 MHz, $CDCl_3$, 213–333 K).

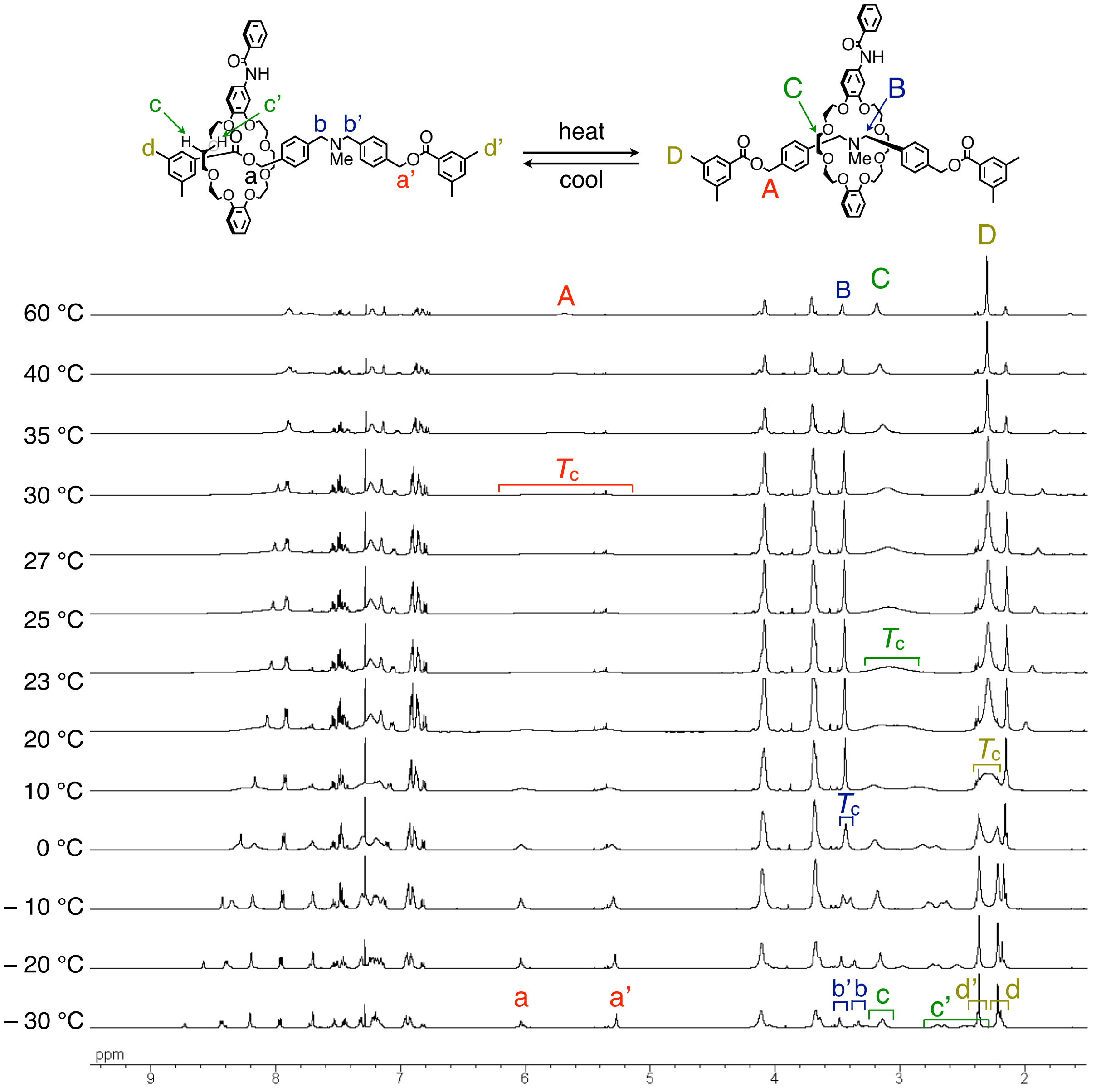

Supplementary Figure S38. VT-^{1}H NMR spectra of **1-Me** (500 MHz, $CDCl_3$, 213–333 K).

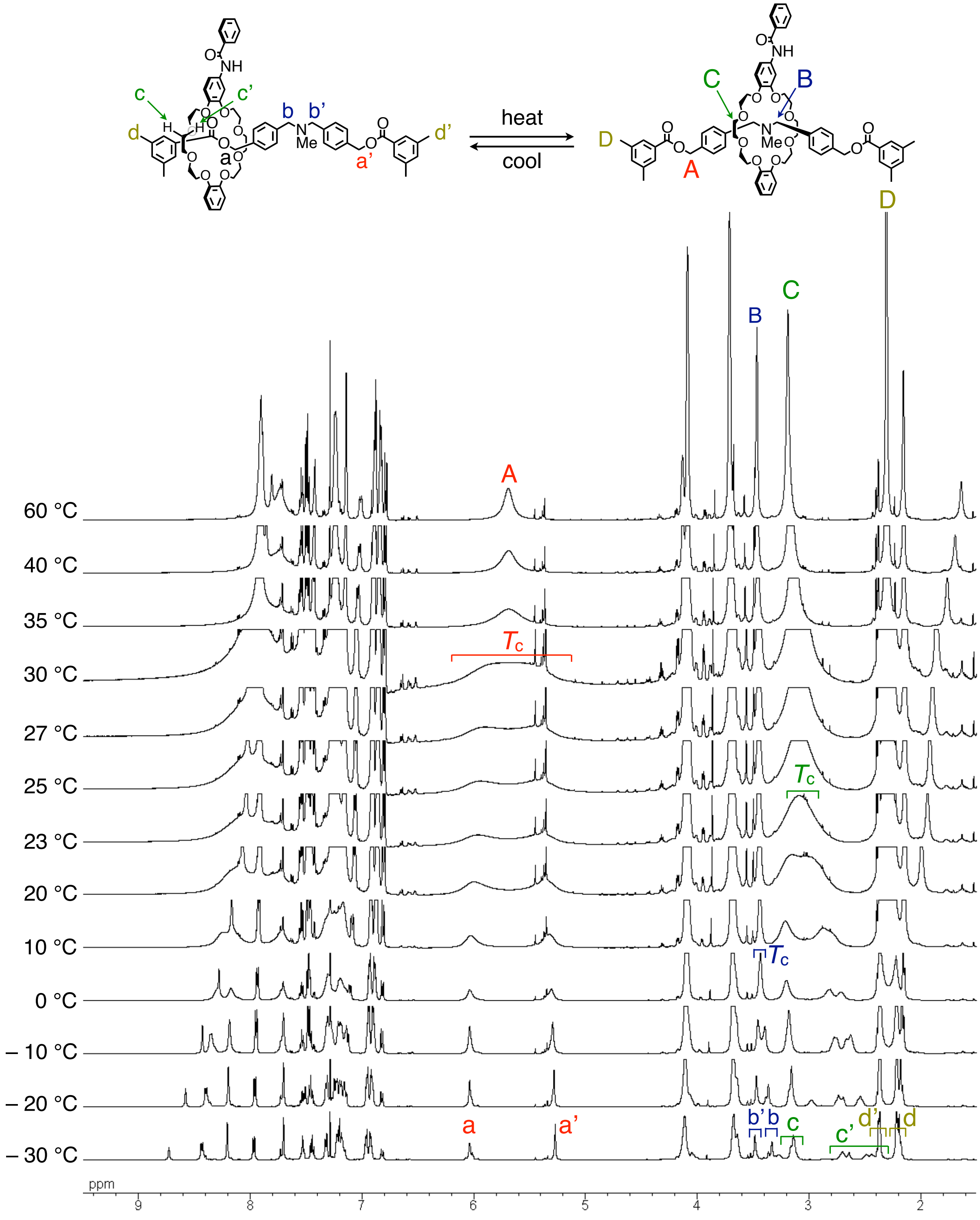

Supplementary Figure S39. Expanded VT-^{1}H NMR spectra of **1-Me** (500 MHz, $CDCl_3$, 213–333 K).

Supplementary Table S1. Kinetic and thermodynamic parameters of **1-Me**.

proton	T_c (K)	Δν	k_c (s^{-1})	E (kJ/mol)	$\Delta G^{\ddagger}$ (kJ/mol)	$\Delta H^{\ddagger}$ (kJ/mol)	$\Delta S^{\ddagger}$ (J/mol • K)
a (500 MHz)	303	383	1053	24.2	56.7	21.7	–115.5
a (400 MHz)	298	326	897		56.1		
b (500 MHz)	273	76.1	209	16.0	54.5	13.8	–149.2
b (400 MHz)	263	58.2	160		53.0		
c (500 MHz)	296	286	787	11.9	56.0	9.49	–157.3
c (400 MHz)	288	250	688		54.8		

Mechanostereoinversion rate constant $k_{\mathrm{inv(c)}}$ and racemization rate constant $k_{\mathrm{rac(c)}}$ at coalescence temperature (T_c) are obtained from equation (1):[1]

$$k_{\mathrm{inv(c)}} = 0.5\ k_{\mathrm{rac(c)}} = \pi\Delta\nu / \sqrt{2} \quad (1)$$

Δν = difference of chemical shift when two peaks are observed separately

Coalescence temperature (T_c) and Δν values are determined from VT-^{1}H NMR spectra of **1-Me** (Supplementary Figures S36–39) and shown in Supplementary Table S1.

Gibbs' activation free energy at coalescence temperature (T_c) for mechanostereoinversion was determined according to Eyring equation (2);

$$\Delta G^{\ddagger} = -RT_c \ln (k_{\mathrm{inv(c)}} h / T_c k_B) \quad (2)$$

$\Delta G^{\ddagger}$: Gibbs' activation free energy

R: gas constant (8.31 m^2 kg s^{-2} K^{-1})

T_c: coalescence temperature

$k_{\mathrm{inv(c)}}$: mechanostereoinversion rate constant at T_c

h: Plank constant (6.63×10^{-34} m^2kg s^{-1})

k_B: Boltzmann constant (1.38×10^{-23} m^2 kg s^{-2} K^{-1})

Gibbs' activation free energy is shown in Supplementary Table S1.

Eyring equation (3) was converted to following equation (4);

$$\ln (k_c / T_c) = \ln (k_B / h) + \Delta S^{\ddagger} / R - \Delta H^{\ddagger} / RT_c \quad (4)$$

$\Delta S^{\ddagger}$: activation entropy

$\Delta H^{\ddagger}$: activation enthalpy

Activation entropy ($\Delta S^{\ddagger}$) and activation enthalpy ($\Delta H^{\ddagger}$) are obtained from the slope value (= $-\Delta H^{\ddagger} / R$), and the intercept value (= $\ln (k_B / h) + \Delta S^{\ddagger} / R$) of Eyring plots (Supplementary Figure S40).

The activation energy for dissociation was determined according to the following equation;

$$k_{rac} = A\exp(-E / RT)$$

It was converted to Arrhenius equation (5);

$$\ln k_{rac} = -E / RT + \ln A \quad (5)$$

E: activation energy

R: gas constant (8.31 m^2 kg s^{-2} K^{-1})

T: absolute temperature

A: frequency factor of Arrhenius equation

Activation energy (E) is obtained from the slope value (= $-E / R$) of Arrhenius plots (Figure S41).

We cannot obtain E, $\Delta H^{\ddagger}$ and $\Delta S^{\ddagger}$ from proton H_c in Supplementary Figures S36–S39 because proton H_c showed almost same coalescence temperatures (T_c) in 400 MHz and 500 MHz NMR spectra.

As for, E, $\Delta H^{\ddagger}$ and $\Delta S^{\ddagger}$ for racemization of **1-Me**, we employed the average values (Table 1 in main text).

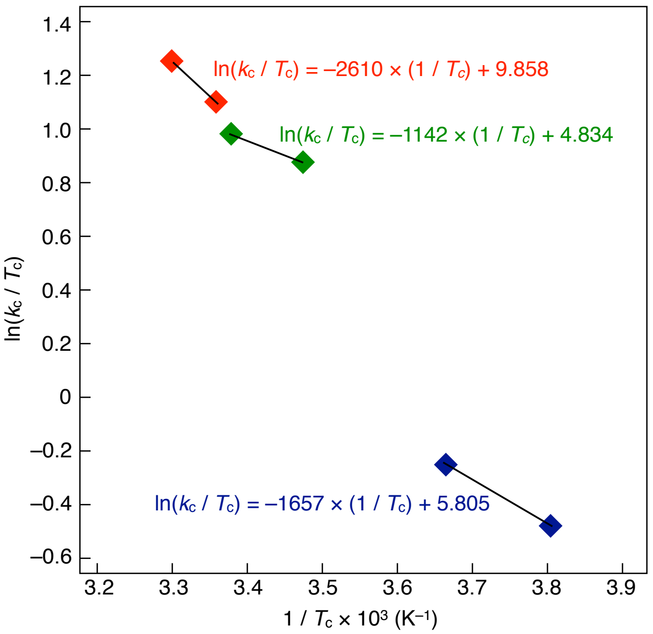

Supplementary Figure S40. Eyring plots for mechanostereoinversion of **1-Me** using calescence of ^{1}H signals of a (red), b (bule) and c (green). Please also see Supplementary Figures S38 and S39.

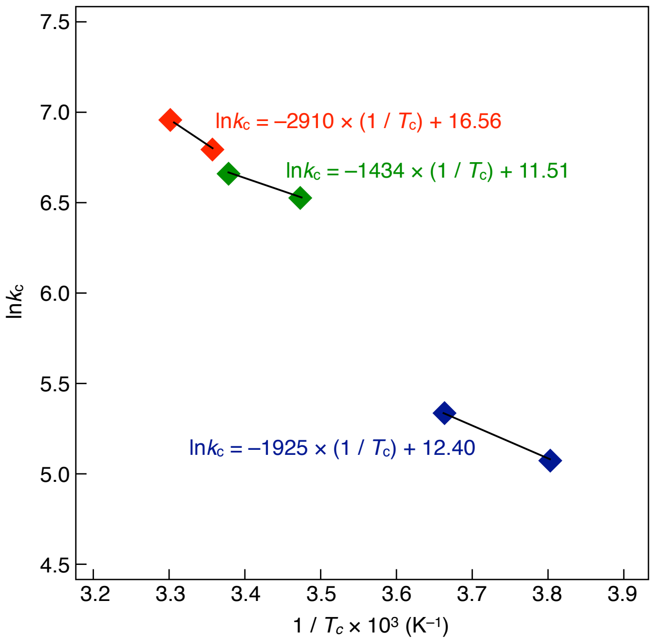

Supplementary Figure S41. Arrhenius plots for mechanostereoinversion of **1-Me** using calescence of ^{1}H signals of a (red), b (bule) and c (green). Please also see Supplementary Figures S38 and S39.

Thermodynamic parameters of racemization of **1-Et** were obtained by CD decay profile (Supplementary Figure S42).

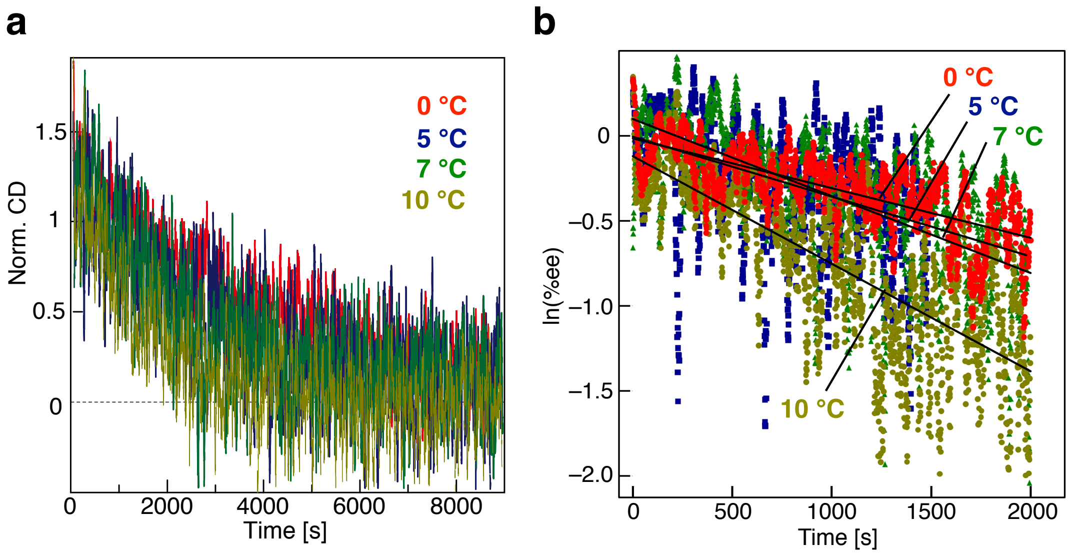

Supplementary Figure S42. (a) Decay profiles and (b) first-order plots of the CD intensities of **1-Et-a** (275 nm) at 0, 5, 7 and 10 °C (0.1 mM, $CHCl_3$).

The racemization rate (k_{rac}) were obtained from equation (6):

$$\ln(\%ee) = -k_{rac}\, t = -2k_{inv}\, t \quad (6)$$

The half-life ($\tau_{1/2}$) of the CD intensity was obtained from equation (7):

$$\tau_{1/2} = \ln 2 / k_{rac} = \ln 2 / 2k_{inv} = 0.347 / k_{inv} \quad (7)$$

The k_{inv} values are obtained from the slope values ($= -k_{inv}$) of first-order plots of the CD intensities (Supplementary Figure S42).

Gibbs' activation free energy for racemization was determined according to Eyring equation (8);

$$\Delta G^{\ddagger} = -RT \ln (k_{inv}h / Tk_B) \quad (8)$$

$\Delta G^{\ddagger}$: Gibbs' activation free energy

R: gas constant (8.31 m^2 kg s^{-2} K^{-1})

T: absolute temperature

k_{inv}: mechanostereoinversion rate constant

h: Plank constant (6.63×10^{-34} m^{2}kg s^{-1})

k_B: Boltzmann constant (1.38×10^{-23} m^2 kg s^{-2} K^{-1})

Eyring equation (8) was converted to following equation (9);

$$\ln (k / T) = \ln (k_B / h) + \Delta S^{\ddagger} / R - \Delta H^{\ddagger} / RT \quad (9)$$

$\Delta S^{\ddagger}$: activation entropy

$\Delta H^{\ddagger}$: activation enthalpy

Additionally, the chiral HPLC profiles of **1-Et-a** at high temperature of 333 K (Figure 4b) and 313 K (Supplementary Figure S33) are typical for those observed in the compounds that undergo racemization in chiral stationary phase. Thus, we also estimated the mechanostereoinversion rate constant k_{inv} from the analysis of HPLC profiles using the DCXplorer MMXVII software (version 3.1.0.1) *i.e.*, dynamic HPLC method.[2] The analysis and determined rate constant k_{inv} are shown in Supplementary Figure S43 and Supplementary Table S2, respectively.

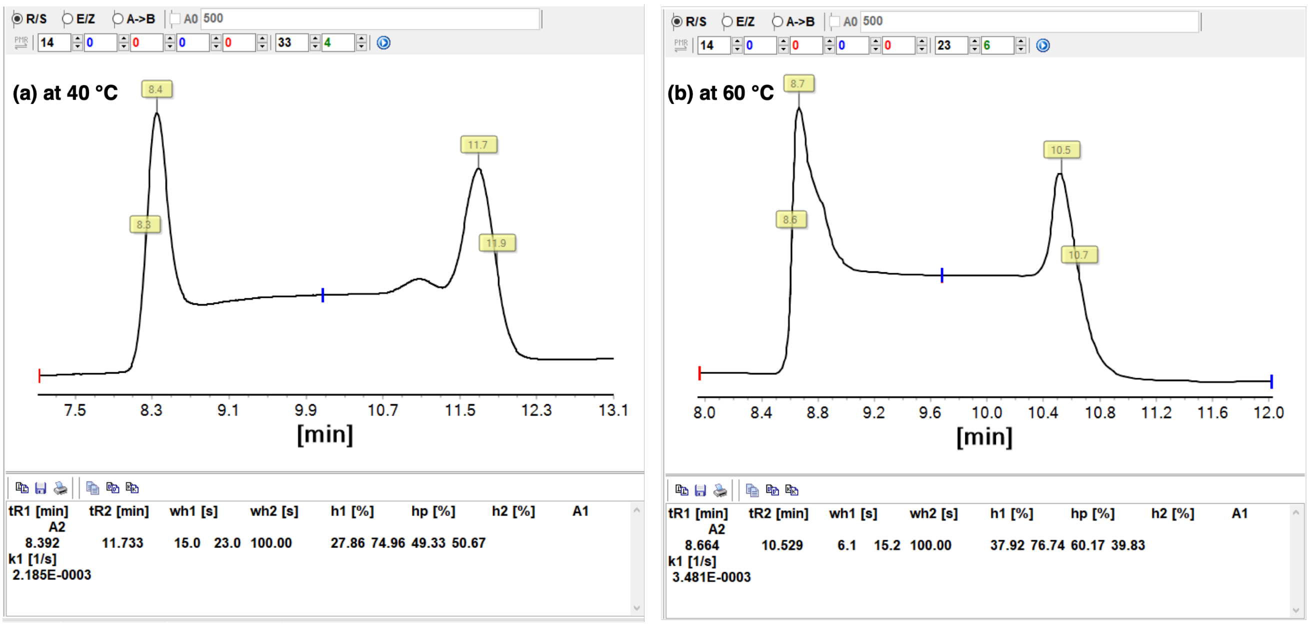

Supplementary Figure S43. Dynamic HPLC analysis of chiral HPLC profiles of **1-Et-*rac*** (CHIRALPAK IA, eluents: n-hexane/$CHCl_3$/Et_2NH = 1/1/0.005, isocratic, flow rate = 1.0 mL/min) at (a) 313 K and (b) 333 K.

From the data obtained from CD decay profiles, activation entropy ($\Delta S^{\ddagger}$) and activation enthalpy ($\Delta H^{\ddagger}$) are obtained from the slope value ($-5545 = -\Delta H^{\ddagger} / R$ in Supplementary Figure S44a), and the intercept value ($5.83 = \ln (k_B / h) + \Delta S^{\ddagger} / R$ in Supplementary Figure S44a) of Eyring plots, and shown in Supplementary Table S3.

From the combined data obtained from CD decay profiles and dynamic HPLC method, activation entropy ($\Delta S^{\ddagger}$) and activation enthalpy ($\Delta H^{\ddagger}$) are obtained from the slope value ($-4777 = -\Delta H^{\ddagger} / R$ in Supplementary Figure S44b), and the intercept value ($3.09 = \ln (k_B / h) + \Delta S^{\ddagger} / R$ in Supplementary Figure S44b) of Eyring plots, and shown in Supplementary Table S3.

The activation energy for dissociation was determined according to the following equation;

$$k_{inv} = A\exp(-E / RT)$$

It was converted to Arrhenius equation (10);

$$\ln k_{inv} = -E / RT + \ln A \quad (10)$$

E: activation energy

R: gas constant (8.31 $m^2\ kg\ s^{-2}\ K^{-1}$)

T: absolute temperature

A: frequency factor of Arrhenius equation

From the data obtained from CD decay profiles, activation energy (E) is obtained from the slope value ($-5077 = -E / R$ in Supplementary Figure S45a) of Arrhenius plots, and shown in Supplementary Table S3.

From the combined data obtained from CD decay profiles and dynamic HPLC method, activation energy (E) is obtained from the slope value ($-5810 = -E / R$ in Supplementary Figure S45b) of Arrhenius plots, and shown in Supplementary Table S3.

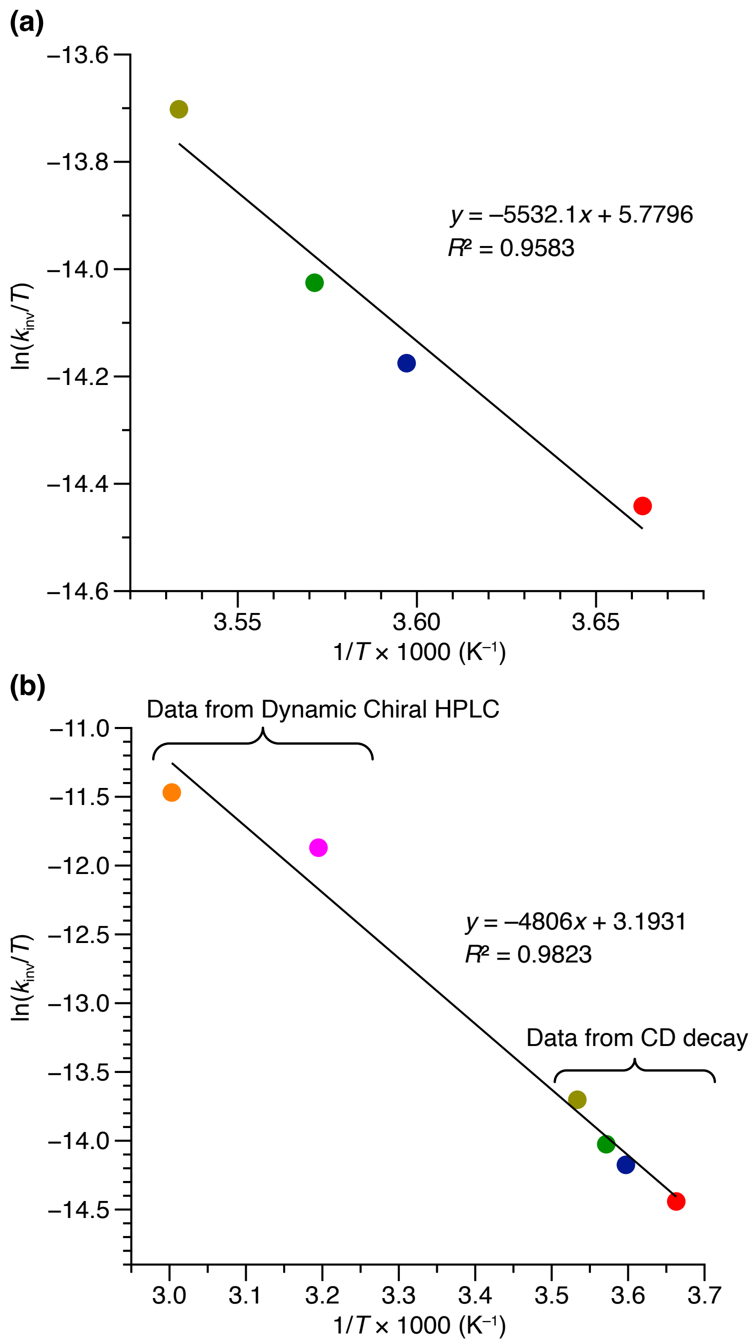

Supplementary Figure S44. (a) Eyring plot for mechanostereoinversion of **1-Et-a** using data obtained from CD decay (in $CHCl_3$) and (b) plot with data obtained from dynamic HPLC (eluents: *n*-hexane/$CHCl_3$/Et_2NH = 1/1/0.005, isocratic, flow rate = 1.0 mL/min).

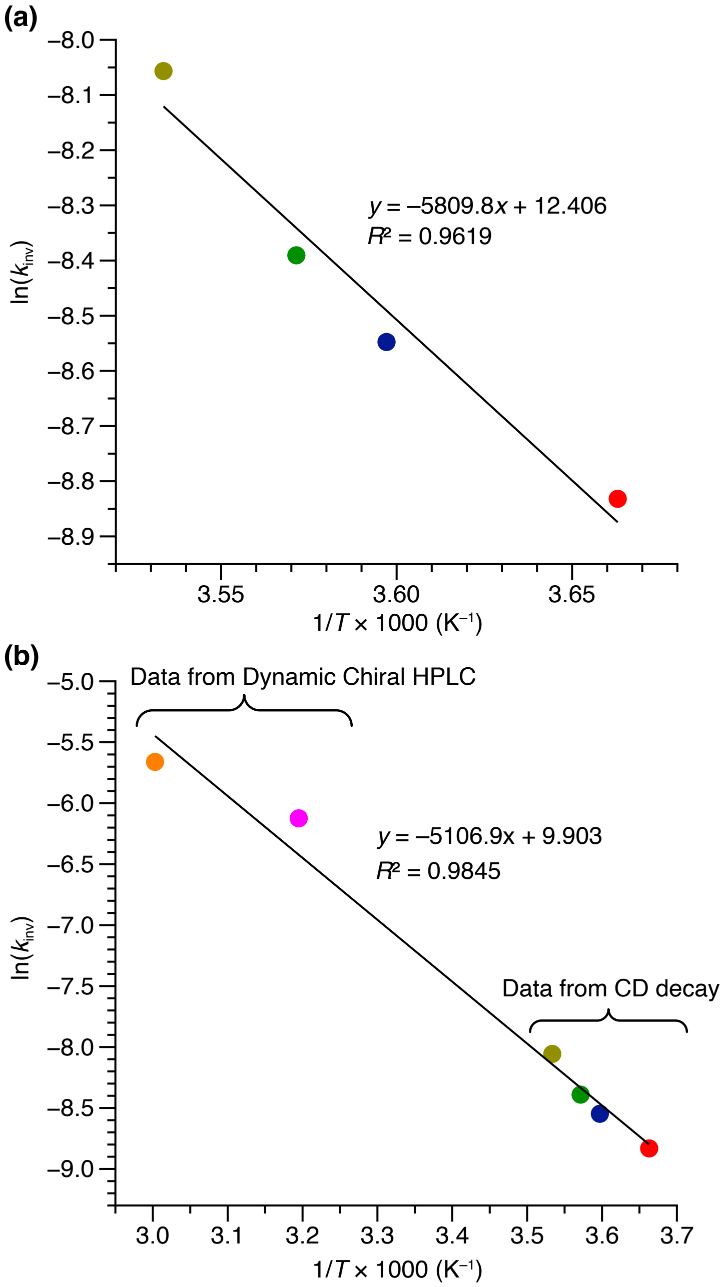

Supplementary Figure S45. (a) Arrhenius plot for mechanostereoinversion of **1-Et-a** using data obtained from CD decay (in $CHCl_3$) and (b) plot with data obtained from dynamic HPLC (eluents: *n*-hexane/$CHCl_3$/Et_2NH = 1/1/0.005, isocratic, flow rate = 1.0 mL/min).

The kinetic and thermodynamic parameters of **1-Et-a** k_{rac}, $\tau_{1/2}$, $\Delta G^‡$, $\Delta S^‡$, $\Delta H^‡$ and E are summarized in Supplementary Tables S2 and S3.

Supplementary Table S2. Kinetic and thermodynamic parameters of **1-Et-a**.

	T (K)	k_{inv} (s^{-1})	$\tau_{1/2}$ (s)	$\Delta G^‡$ (kJ/mol)
from CD decay	273	1.46×10^{-4}	2.37×10^{3}	86.7
	278	1.94×10^{-4}	1.79×10^{3}	87.6
	280	2.27×10^{-4}	1.53×10^{3}	87.9
	283	3.17×10^{-4}	1.09×10^{3}	88.1
from Dynamic HPLC	313	2.19×10^{-3}	1.58×10^{2}	92.7
	333	3.48×10^{-3}	9.96×10^{1}	97.5

Supplementary Table S3. Thermodynamic parameters of **1-Et-a**.[a]

	E (kJ/mol)	$\Delta H^‡$ (kJ/mol)	$\Delta S^‡$ (J/mol•K)
**1-Et** (from CD decay only)	48.2	46.1	–61.5
**1-Et** (from CD decay and Dynamic HPLC)	48.4	39.7	–38.7

[a] The values of thermodynamic parameters of **1-Et** determined from CD decay only and from combined data with dynamic HPLC are approximately consistent. But in the main text, we showed the thermodynamic parameters of **1-Et** determined from CD decay only, because the solvent condition are largely different in these methods.

The kinetic and thermodynamic parameters of **1-Ac-a** (k_{inv}, $\tau_{1/2}$, $\Delta G^{\ddagger}$, $\Delta S^{\ddagger}$, $\Delta H^{\ddagger}$ and E) are obtained by the same way as **1-Et-a**. CD decay profiles, Eyring plots and Arrhenius plots are shown in Supplementary Figures S45, S47 and S48 respectively. Time dependent CD spectra of **1-Ac-a** is shown in Supplementary Figure S46. The kinetic and thermodynamic parameters of **1-Ac-a** are summarized in Supplementary Tables S4 and S5.

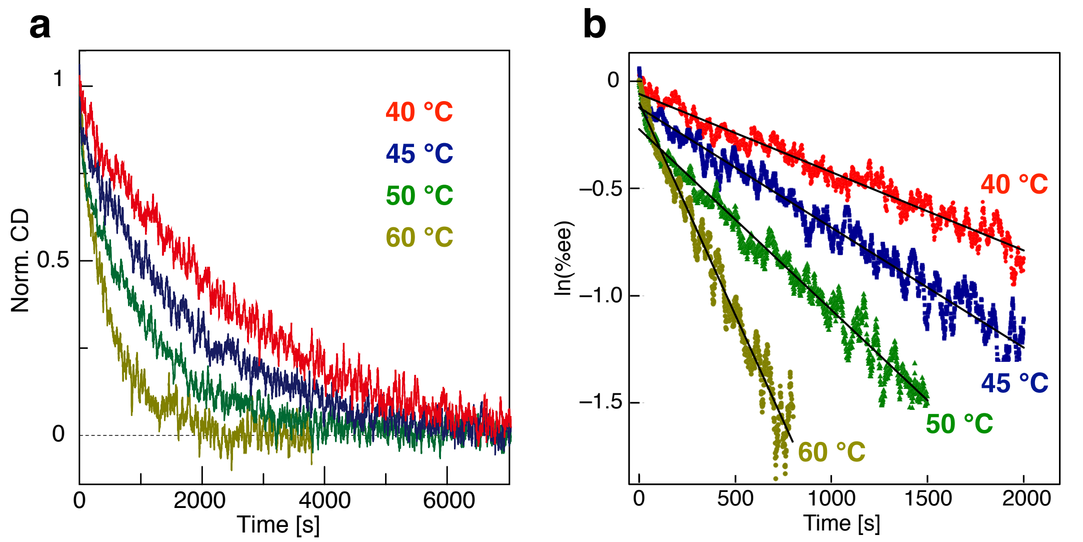

Supplementary Figure S46. (a) Decay profiles and (b) first-order plots of the CD intensities of **1-Ac-a** (275 nm) at 40, 45, 50, and 60 °C (0.1 mM, $CHCl_3$).

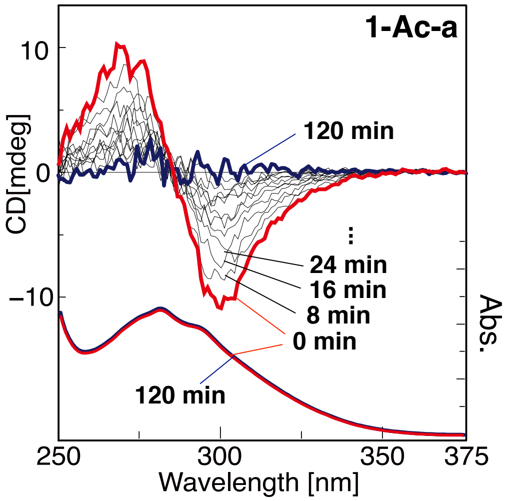

Supplementary Figure S47. Time dependent CD spectra of **1-Ac-a** at 313 K (0.1 mM, $CHCl_3$, 313 K).

Activation entropy ($\Delta S^{\ddagger}$) and activation enthalpy ($\Delta H^{\ddagger}$) are obtained from the slope value (–8491 = –$\Delta H^{\ddagger}$ / R in Supplementary Figure S48a), and the intercept value (12.8 = ln (k_B / h) + $\Delta S^{\ddagger}$ / R in Supplementary Figure S44a) of Eyring plots, and shown in Supplementary Table S5. Activation energy (E) is obtained from the slope value (–8814 = –E / R in Supplementary Figure S45a) of Arrhenius plots, and shown in Supplementary Table S5.

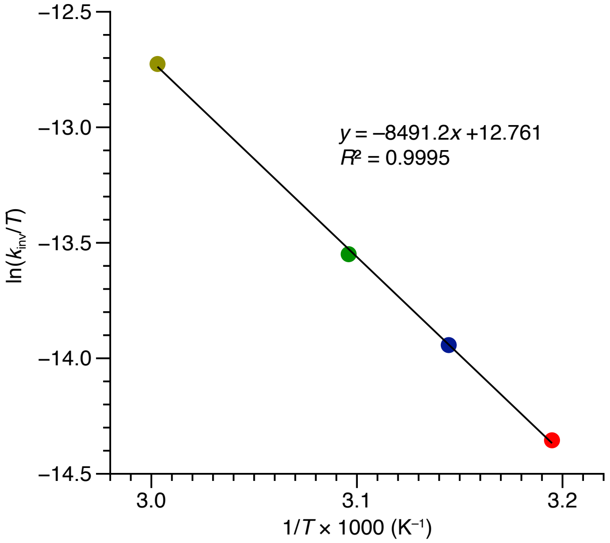

Supplementary Figure S48. Eyring plots for mechanostereoinversion of **1-Ac-a**.

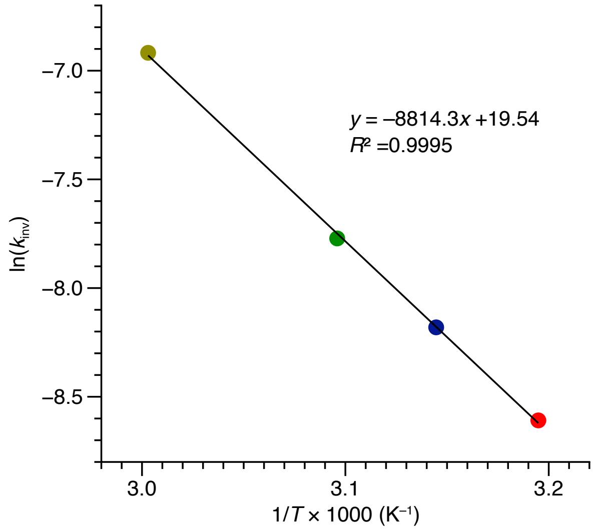

Supplementary Figure S49. Arrhenius plots for mechanostereoinversion of **1-Ac-a**.

Supplementary Table S4. Kinetic and thermodynamic parameters of **1-Ac-a**.

T (K)	k_{inv} (s^{-1})	$\tau_{1/2}$ (s)	$\Delta G^{\ddagger}$ (kJ/mol)
313	1.83×10^{-4}	1.90×10^{3}	99.1
318	2.80×10^{-4}	1.24×10^{3}	99.6
323	4.22×10^{-4}	8.22×10^{2}	100.1
333	9.90×10^{-4}	3.50×10^{2}	101.0

Supplementary Table S5. Thermodynamic parameters of **1-Ac-a**.

	E (kJ/mol)	$\Delta H^{\ddagger}$ (kJ/mol)	$\Delta S^{\ddagger}$ (J/mol•K)
**1-Ac**	73.2	70.6	–119

Schematic Illustration

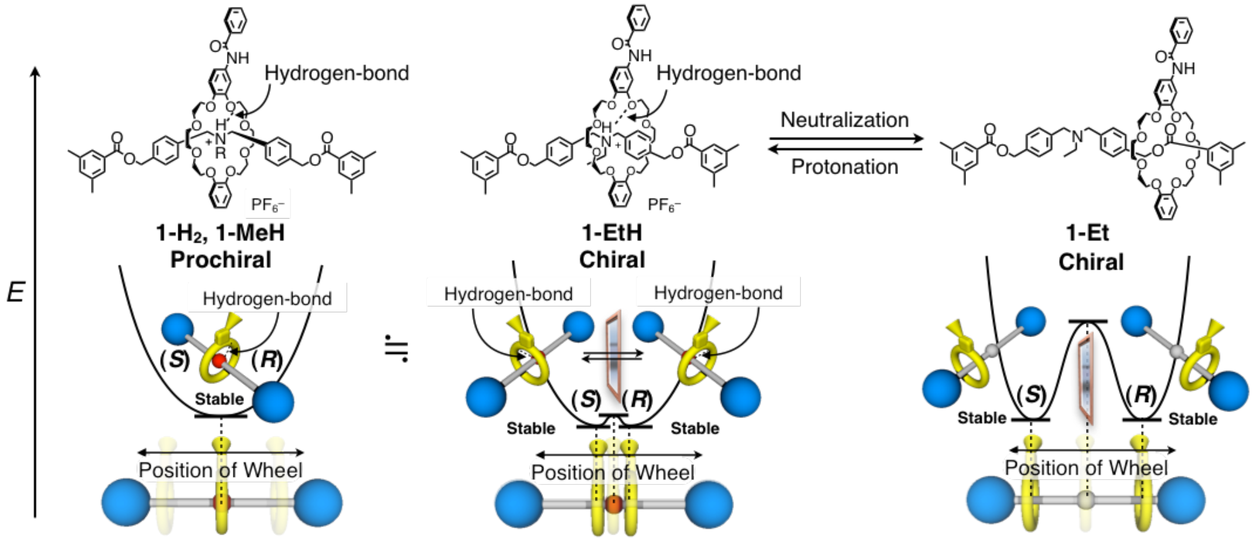

Supplementary Figure S50. Schematic illustration of structures and thermodynamic diagrams of rotaxanes. Upon protonation to **1-Et**, the position of wheel component, stable structure and thermodynamic diagrams changed dramatically. The wheel component of **1-EtH** is fixed at the center of wheel component by hydrogen bonding, and both stable conformations of **1-EtH** have very low mechanostereoinversion energy and the optical resolution is impossible. Thus, the structure of **1-EtH** is almost same as that of prochiral **1-H$_2$**, and can be virtually regarded as a prochiral species.

Supplementary References

1) (a) Kaplan, J. I.; Fánkel, G. in *NMR of Chemically Exchanging Systems*, Academic Press, New York, **1980**. (b) Oki, M. in Methods in *Stereochemical Analysis*, Vol. 4, Verlag, Weinheim, **1985**.
2) Trapp, O (2006). Unified equation for access to rate constants of first-order reactions in dynamic and on-column reaction chromatography. *Anal. Chem. 78*, 189–198.
